# *HER2* G776S mutation promotes oncogenic potential in colorectal cancer cells when accompanied by loss of APC function

**DOI:** 10.1038/s41598-022-13189-y

**Published:** 2022-06-02

**Authors:** Yosuke Mitani, Shinya Ohashi, Osamu Kikuchi, Yukie Nakai, Tomomi Ida, Ayaka Mizumoto, Yoshihiro Yamamoto, Tomoki Saito, Shigeki Kataoka, Junichi Matsubara, Atsushi Yamada, Masashi Kanai, Shigemi Matsumoto, Hiroaki Sakai, Kiyotsugu Yoshikawa, Eijiro Nakamura, Manabu Muto

**Affiliations:** 1 Department of Therapeutic Oncology, Kyoto University Graduate School of Medicine, 54 Shogoin Kawahara-cho, Sakyo-ku, Kyoto, 606-8507 Japan; 2 DSK Project, Medical Innovation Center, Kyoto University Graduate School of Medicine, 54 Shogoin Kawahara-cho, Sakyo-ku, Kyoto, 606-8507 Japan; 3 Department of Clinical Pharmacy Faculty of Pharmaceutical Sciences, Doshisha Women’s College of Liberal Arts, Keisui-kan, 97-1 Minami-hodate Kodo, Kyotanabe, Kyoto W604610-0395 Japan; 4 Department of Urology, National Cancer Center Hospital, 5-1-1 Tsukiji, Chuo-ku, Tokyo, 104-0045 Japan

**Keywords:** Cancer, Cell biology, Genetics

## Abstract

Clinical cancer genome sequencing detects oncogenic variants that are potential targets for cancer treatment, but it also detects variants of unknown significance. These variants may interact with each other to influence tumor pathophysiology, however, such interactions have not been fully elucidated. Additionally, the effect of target therapy for those variants also unclarified. In this study, we investigated the biological functions of a *HER2* mutation (G776S mutation) of unknown pathological significance, which was detected together with *APC* mutation by cancer genome sequencing of samples from a colorectal cancer (CRC) patient. Transfection of the *HER2* G776S mutation alone slightly increased the kinase activity and phosphorylation of HER2 protein, but did not activate HER2 downstream signaling or alter the cell phenotype. On the other hand, the *HER2* G776S mutation was shown to have strong oncogenic potential when loss of APC function was accompanied. We revealed that loss of APC function increased Wnt pathway activity but also increased RAS–GTP, which increased ERK phosphorylation triggered by *HER2* G776S transfection. In addition, afatinib, a pan-HER tyrosine kinase inhibitor, suppressed tumor growth in xenografts derived from *HER2* G776S-transfected CRC cells. These findings suggest that this *HER2* mutation in CRC may be a potential therapeutic target.

## Introduction

In recent years, the development of next-generation sequencing (NGS) has enabled the detailed analysis of the cancer genome in cancer patients, and targeting of oncogenic driver alterations has been shown to be effective in several types of cancers^1–4^. One of the possible targets among oncogenic driver alterations is the human epidermal growth factor receptor 2 (*HER2*)^5,6^, also known as *ERBB2*, which is a member of the epidermal growth factor receptor (EGFR) family of homologous transmembrane receptor tyrosine kinases^7^. HER2-targeted therapies have improved outcomes for cancer patients with HER2 gene amplification and overexpression^8,9^. Somatic point mutations in *HER2* can also be detected by NGS in a number of cancers^10,11^. Several major *HER2* mutations, such as V777L in breast cancer^12^ and *HER2* exon 20 insertion mutations in lung cancer^13^, have been shown to be driver mutations that promote oncogenicity through constant phosphorylation of HER2 and are expected to be therapeutic targets.

In colorectal cancer (CRC), somatic point mutations in *HER2* are detected in 4–6% of cases^6,14^, most of which are diverse single nucleotide variants^6,10^. V777L and V842I are the well-known mutations in CRC^5^, and these mutations have been shown to be associated with phosphorylation of extracellular signal-regulated kinase (ERK) and increased anchorage-independent cell growth activity^15^. These previous reports indicate the significance of *HER2* mutations in CRC; however, numerous other *HER2* mutations remain variants of unknown significance. Moreover, clinical NGS often detects multiple gene variants in a single patient^3,16^, and these variants may interact with each other and affect cancer cell tumorigenesis. However, the interactions between gene variants are not fully understood.

In this study, we focused on a patient with CRC treated in our hospital whose *HER2* G776S mutation (c.2326G > A, p.Gly776Ser) and an *APC* nonsense mutation were detected by clinical NGS. *HER2* G776S is located in the tyrosine kinase domain in HER2 (amino acid 721–975). This domain has been found to have more frequent mutations compared with other domains^6,17^. Several mutations such as A775_G776insYVMA and G776 > VC have been annotated as activating variants in non-small-cell lung carcinoma^18^. However, this G776S mutation is rare, and its significance is not fully understood. Here, we examined the oncogenic function and treatment sensitivity of the *HER2* G776S mutation and assessed the interaction between the *HER2* G776S mutation and loss of APC function.

## Results

### NGS-based multiplex gene assay for molecular profiling analysis and variant annotation in a CRC patient

**Figure 1:**
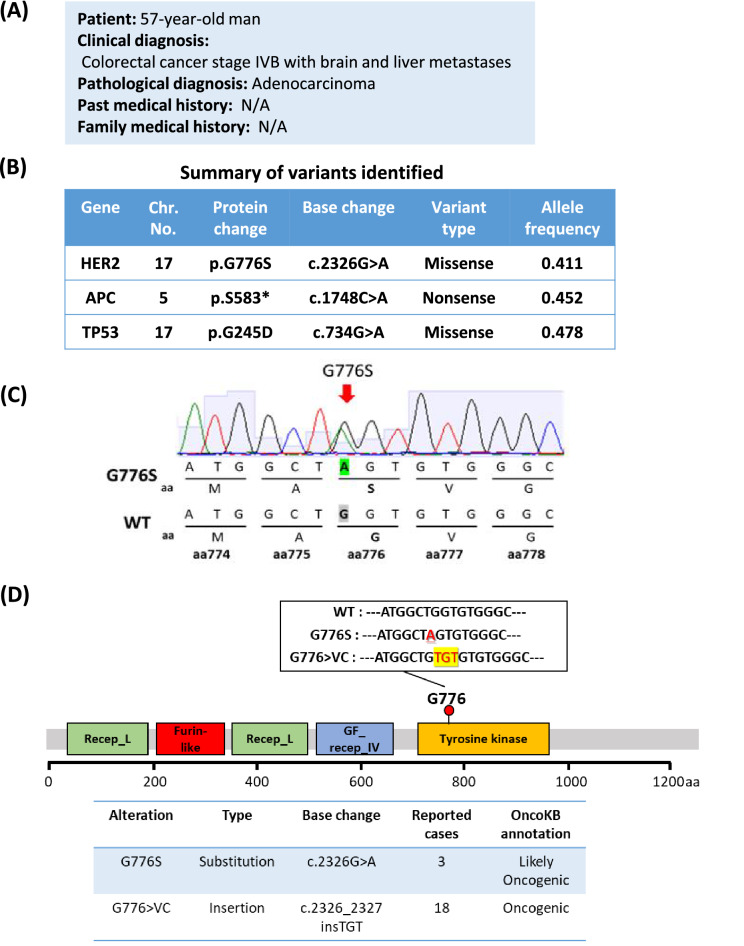
Genetic characteristics of a case of colorectal cancer harboring *HER2* G776S mutation detected by clinical NGS. (**A**) Summary of the patient's clinical profile. (**B**) Comprehensive genomic analysis report of the cancer of the patient. Positive biomarkers are shown. (**C**) Sanger sequencing of a part of *HER2* gene of the cancer genome DNA. The amino acid (a.a.) sequence from 774 to 778 is shown. The sequence of the corresponding wild-type (WT) site is also presented. The red arrow indicates the mutation position of G776S. (**D**) The mutation position of G776 in HER2 protein domains based on the COSMIC database. The nucleotide sequences of the WT, G776S and G776 > VC are described. The underlined part indicates a substitution mutation and the yellow background site indicates a short insertion. Recep_L, receptor L domain; Furin-like, furin-like cysteine-rich region; GF_recep_IV, growth factor receptor domain IV. In the lower part of the figure, the number of reports in the COSMIC database and annotations from OnkoKB (https://www.oncokb.org/) are shown. The data are current as of April 2021.

The patient, a 57-year-old man, was diagnosed with colorectal adenocarcinoma with liver metastasis (Fig. 1A). After resection of the primary lesion, eight courses of chemotherapy with mFOLFOX6 plus cetuximab (anti-EGFR antibody) were performed. Subsequently, a right hepatic lobectomy was performed, but recurrence occurred in the liver, lung and brain, and the patient was referred to Kyoto University Hospital for clinical NGS examination.

As shown in Fig. 1B, three somatic mutations, *HER2* G776S mutation, *APC* nonsense mutation, and *TP53* missense mutation, were detected from his liver metastasis specimen by the NGS-based multiplex gene assay. Other mutations related to the HER2 signaling pathway (*KRAS*, *NRAS*, *BRAF*, and *PIK3CA*) were not detected. We also confirmed the *HER2* G776S mutation in this specimen using Sanger sequencing (Fig. 1C). *HER2* copy number amplification was not detected by real-time quantitative PCR (Supplementary Fig. 1).

According to the COSMIC database (v91; http://cancer.sanger.ac.uk/cosmic/), the amino acid position G776 is in the kinase domain of HER2, and several mutations in this region, such as G776 > VC, have been registered as oncogenic genes (Fig. 1D). G776S is a rare mutation that has been detected in three cases: two cases of gastric cancer and one case of urothelial tract cancer. However, its significance has not been elucidated.

### Effect of *HER2* G776S mutation on kinase activity and phosphorylation and its evaluation in classical cell-based transfection assays

**Figure 2:**
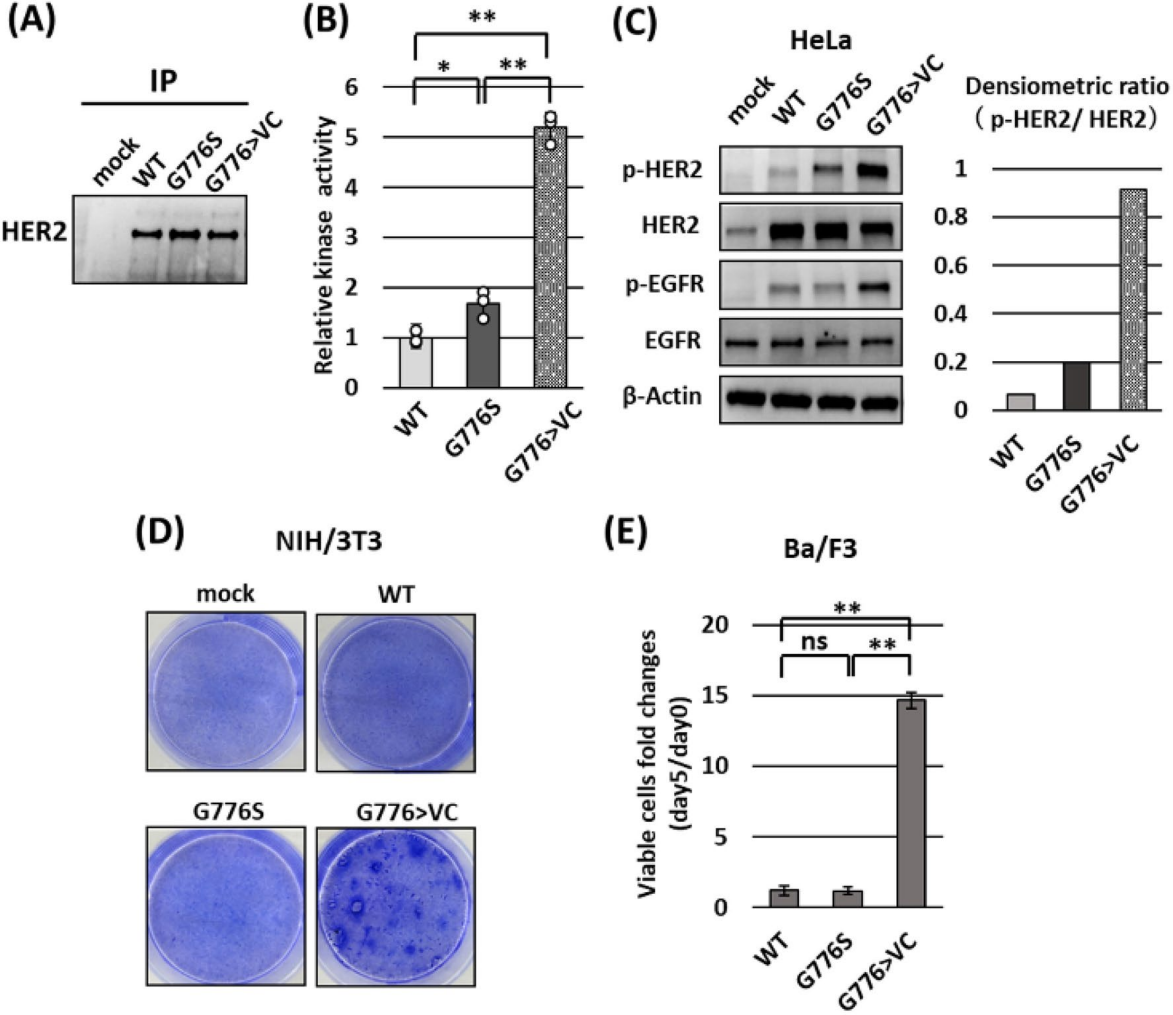
Effects of *HER2* mutations on kinase activity and phosphorylation, and their functional evaluation in classical cell-based transfection assays. (**A**) Western blot results showing recombinant proteins of *HER2* WT and mutants (G776S and G776 > VC). HeLa cells were transiently transfected with the *HER2* expression vectors, and 48 h after the transfection, the cells were lysed and purified by immunoprecipitation using HER2 antibody. (**B**) Measurement of the kinase activity of 100 ng of recombinant HER2 protein using the kinase assay. Kinase activity was measured in triplicate, and the data were standardized to the mean HER2 WT activity. One-way ANOVA: *P* < 0.01, *P < 0.05, **P < 0.01. (**C**) Western blot results for the expression and phosphorylation of HER2 and EGFR in HeLa cells transfected with the *HER2* expression vectors. β-Actin served as an internal control. The bar graph indicates the normalized ratios of densitometric values of phosphorylated to total HER2 protein. (**D**) Focus formation assays using NIH/3T3 cells stably transfected with the indicated plasmids. (**E**) Ba/F3 transformation assays using Ba/F3 (interleukin 3-dependent cells) stably transfected with the indicated plasmids. The bar graph shows the fold changes in number of viable cells. ns, not significant. One-way ANOVA: *P* < 0.01, **P < 0.01 (*n* = 3).

We established plasmid expression vectors containing human cDNA encoding full-length *HER2* wild-type (WT) (Supplementary Fig. 2A), mutant *HER2* G776S, and G776 > VC as a positive control (Supplementary Fig. 2B). To evaluate the effect of *HER2* G776S on kinase activity, we transiently transfected the *HER2* WT or *HER2* mutant expression vectors into HeLa cells, which do not have oncogenic variants in RTK-RAS-ERK pathway or Wnt/β-actin pathway. (Supplementary Table 1). We confirmed by immunoprecipitation that HER2 proteins were successfully generated in the transfected cells (Fig. 2A).

We measured their kinase activity toward peptide substrates. The kinase activity was significantly higher for HER2 G776S protein than WT protein but was less than that of *HER2* G776 > VC (Fig. 2B). We assessed the phosphorylation of HER2 and EGFR, which is one of the dimerization partners of HER2, in WT-, G776S- and G776 > VC-transfected HeLa cells. As shown in Fig. 2C, G776S and G776 > VC transfection increased the phosphorylation of both HER2 and EGFR. However, similar to the results for kinase activity, the degree of HER2 and EGFR phosphorylation was weaker for G776S transfection than for G776 > VC transfection.

Next, we evaluated the oncogenic potential of *HER2* G776S using classical cell-based transfection assay using murine cell lines, NIH/3T3 focus formation assay^19^, and Ba/F3 transformation assay^4,20^. NIH/3T3 cells acquired the focus-forming ability by transfection with G776 > VC but not with G776S (Fig. 2D). Similarly, Ba/F3 cells exhibited IL3-independent proliferation upon transfection with G776 > VC but not G776S (Fig. 2E). These findings suggest that HER2 G776S had weaker phosphorylation capacity and kinase activity than the known HER2 mutation and did not promote the transformed phenotype in the transfection assays using murine cells.

### Differences in the effects of *HER2* G776S mutation on activation of the *HER2* signaling pathway between *APC*-intact and *APC*-mutant cells

**Figure 3:**
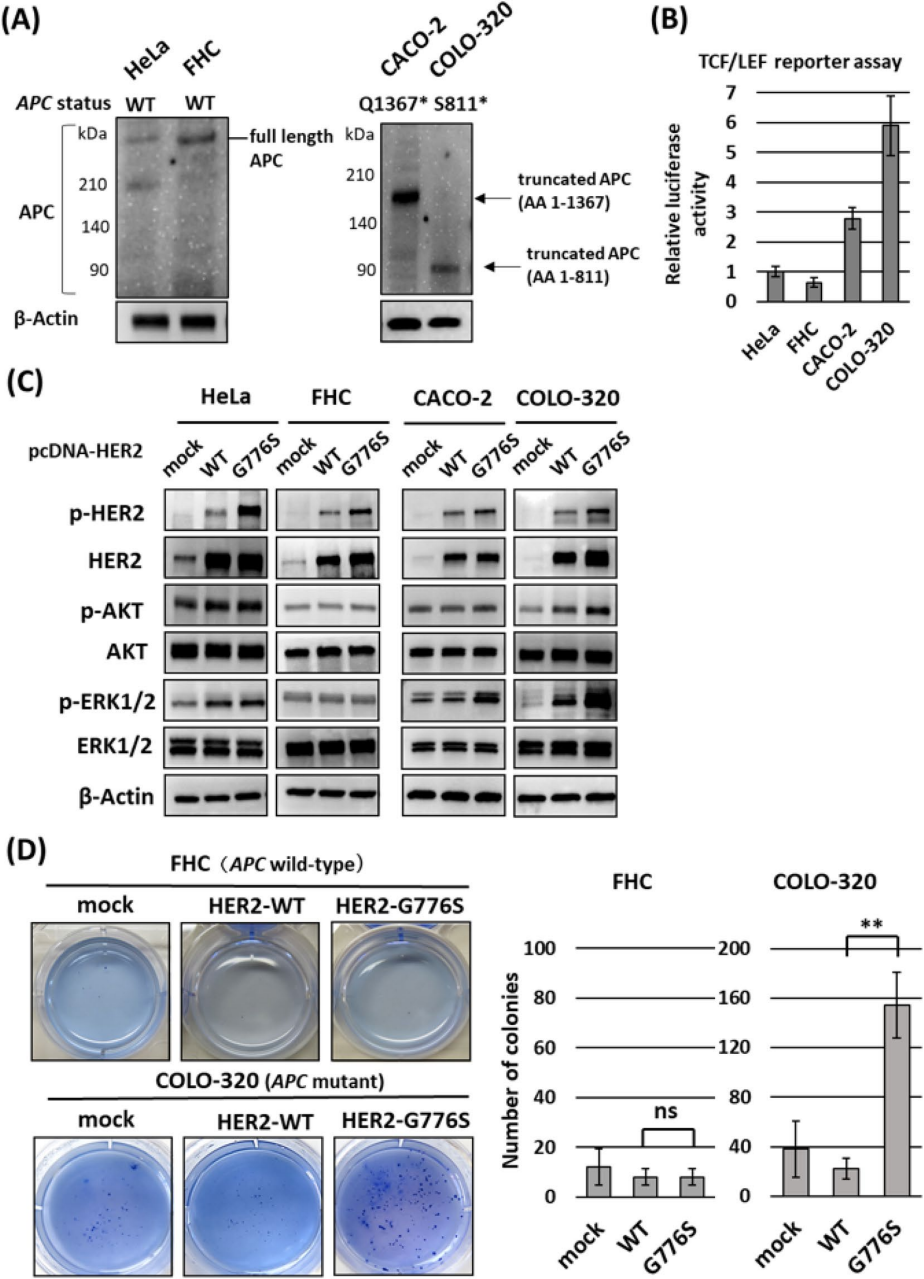
Effects of *HER2* G776S mutation on the *HER2* signaling pathway and anchorage-independent growth in several cell lines. (**A**) Detection of APC proteins in each cell by Western blotting. Full-length APC was detected with APC antibody (#2504, Cell Signaling Technology), and truncated APC was detected with APC antibody (sc-9998, Santa Cruz Biotechnology) raised against the N-terminus of APC. (**B**) The activity of the Wnt/β-catenin signaling pathway measured using the TCF/LEF luciferase reporter assay. The assay was performed in triplicate and the results were standardized to the mean of the activity of HeLa cells. (**C**) Phosphorylation and expression of HER2 and the downstream signaling in WT *HER2*- or *HER2* G776S-transfected HeLa and colon cells (FHC, CACO-2 and COLO-320). (**D**) Soft agar colony-forming assays showing the effects of stable transfection with *HER2* WT or *HER2* G776S in FHC (WT *APC*) and COLO-320 (mutant *APC*) cells. The cells were seeded into six-well plates in triplicate, and the number of colonies per well was counted. ns, not significant. **P < 0.01 *HER2* WT vs *HER2* G776S.

To assess the effect of the *HER2* G776S mutation on the *HER2* downstream signaling pathway in human cancer cells, we transfected *HER2* WT or *HER2* G776S expression vectors into several human cell lines: HeLa (cervical cancer cells), FHC (fetal human colon epithelial cells), CACO-2 (colorectal cancer cells) and COLO-320 cells (colorectal cancer cells). As described in the Methods section, these cell lines have no *HER2* mutations or amplifications and no mutation in the *HER2* downstream signals genes, *KRAS, NRAS, BRAF* or *PIK3CA* (Supplementary Table 1). HeLa and FHC cells are *APC* WT, and CACO-2 and COLO-320 harbor *APC* mutations. Using Western blotting, we consistently detected full-length APC in HeLa and FHC cells, and truncated APC in CACO-2 and COLO-320 cells (Fig. 3A). Using the TCF/LEF reporter assay, we confirmed that the Wnt activity was relatively higher in CACO-2 and COLO-320 cells than in the other two cell lines (HeLa and FHC cells) (Fig. 3B).

As shown in Fig. 3C, G776S transfection increased the amount of phosphorylated HER2 compared with WT transfection in all cell lines. Of note, G776S transfection increased the phosphorylation of the HER2 downstream signaling proteins, AKT and ERK1/2, in CACO-2 and COLO-320 cells (with mutant APC) but not in HeLa and FHC cells (with WT *APC*). Next, we established stably transfected FHC and COLO-320 cells and examined the effects of *HER2* G776S transfection on cell proliferation and anchorage-independent cell growth activity. Transfection with *HER2* WT or G776S did not affect cell proliferation in FHC or COLO-320 cells (Supplementary Fig. 3). By contrast, G776S transfection significantly increased anchorage-independent cell growth activity in COLO-320 cells (mutant APC) but not in FHC cells (WT *APC*). WT transfection did not alter anchorage-independent growth in either cell line (Fig. 3D).

Based on these findings, we hypothesize that the *HER2* G776S mutation activates HER2 downstream signaling and acts as an oncogenic driver mutation in cells with *APC* mutations but is not sufficient to be oncogenic in cells with WT *APC*.

### APC function interference in the effect of the *HER2* G776S mutation on the *HER2* signaling pathway activation

**Figure 4:**
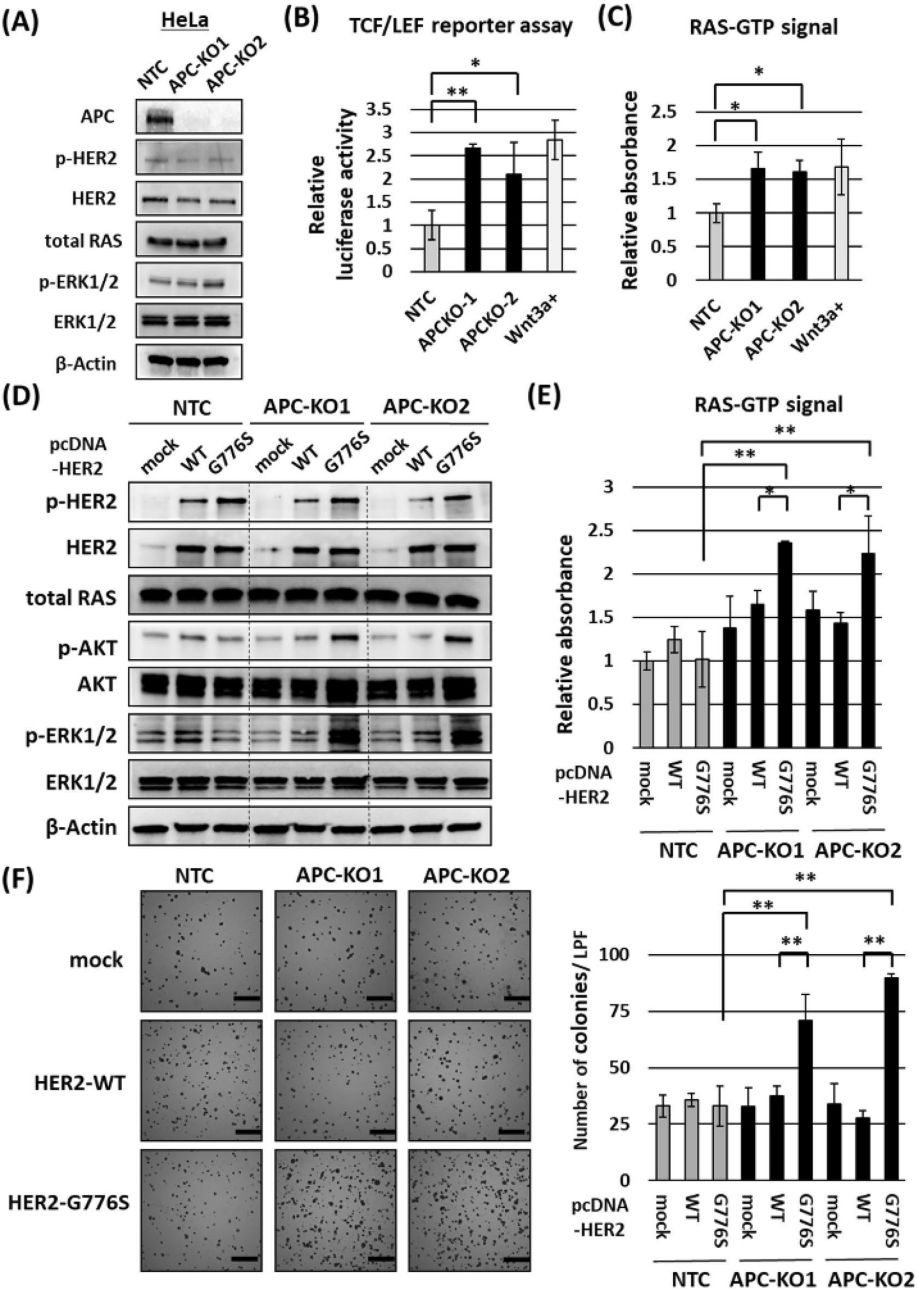
Effects of *APC* KO on HER signaling in HeLa cells (with WT *APC*). (**A**) Confirmation of *APC* KO and its effect on the HER2–ERK pathway in *APC*-KO HeLa cells using Western blotting. β-Actin served as a loading control. (**B**) Activity of the Wnt/β-catenin signaling pathway measured using the TCF/LEF luciferase reporter assay. Nontargeting control cells (NTC) incubated with Wnt3a (100 ng/ml) for 24 h were used as a positive control (rightmost bar). The data were standardized to the mean of the activity of NTC cells. One-way ANOVA: P < 0.01, *P < 0.05, **P < 0.01 vs NTC cells (*n* = 3). (**C**) Measurement of activated RAS (RAS–GTP) using the G-LISA assay. NTC plus Wnt3a (100 ng/ml) was used as a control (rightmost bar). The data were standardized to the mean of the activity of NTC cells. One-way ANOVA: P < 0.05, *P < 0.05 vs NTC cells (*n* = 3). (**D**) Western blot results showing the effects of *HER2* WT or *HER2* G776S transfection on the HER2 signaling pathway in *APC*-KO cells. *APC*-KO cells or NTC cells were transfected with mock or *HER2* expression vectors, and the experiments were performed 48 h after transfection. β-Actin served as a loading control. (**E**) Measurement of RAS-GTP using the G-LISA assay performed 48 h after *HER2* expression vector transfection. The data were standardized to the mean of the activity of NTC cells transfected with mock vectors. Two-way ANOVA: interaction *P* < 0.01, *P < 0.05, **P < 0.01 (*n* = 3). (**F**) Colony-forming assay results showing the effects of stable transfection with *HER2* WT or *HER2* G776S in *APC*-KO cells. The number of colonies was counted in six random low-power fields. Scale bar, 200 μm. Two-way ANOVA: interaction *P* < 0.01, **P < 0.01.

To evaluate the effects of loss of APC function on the *HER2* signaling pathway, we used CRISPR-Cas9 to establish *APC*-KO cells. We tried to establish *APC*-KO cells using colon cell lines, such as FHC, but experienced difficulty because of the extremely poor infection efficiency of the lentivirus; therefore, we established *APC*-KO HeLa cells. As described in Supplementary Table 1, HeLa cells have no oncogenic variants in the RTK–RAS–ERK pathway or Wnt/β-actin pathway. As shown in Fig. 4A, we confirmed the loss of full-length APC protein in *APC*-KO cells. We used the TCF/LEF reporter assay to confirm that the Wnt/β-catenin signaling pathway was significantly activated in *APC*-KO cells compared with nontargeting control (NTC) cells (Fig. 4B).

To examine the effects of *APC* deficiency on the HER2 signaling pathway, we assessed the amount and phosphorylation of HER2 and downstream signal proteins, including detection of RAS–GTP (activated RAS), in *APC*-KO cells and NTC cells. *APC* KO significantly increased the amount of RAS–GTP (Fig. 4C) and slightly increased phosphorylated ERK 1/2 (Fig. 4A), whereas there was no apparent change in the amount or phosphorylation of other proteins of the HER2 signaling pathway (HER2, CRAF, MEK and AKT) (Fig. 4A and Supplementary Fig. 4A). These results suggest that *APC* KO increases RAS–GTP as well as the activity of the Wnt pathway without affecting phosphorylation of HER2 and other downstream signals.

To assess the effects of activation of the *HER2* signaling pathway in G776S-transfected *APC*-KO cells, we next transiently transfected the *HER2* WT or *HER2* G776S expression plasmid vector into *APC*-KO and NTC cells. As shown in Fig. 3D, *HER2* G776S transfection massively increased the phosphorylation of downstream proteins in the HER2 pathway, especially ERK1/2, in *APC*-KO cells. By contrast, G776S transfection did not affect AKT or ERK1/2 phosphorylation in NTC cells (Fig. 4D). G776S transfection and KO of APC synergistically increased RAS-GTP. (Interaction P < 0.01, two-way ANOVA) (Fig. 4E). Moreover, G776S transfection in *APC*-KO cells significantly increased anchorage-independent growth, whereas growth was not affected by mock or HER2 WT transfection in *APC*-KO cells (Fig. 4F).

**Figure 5:**
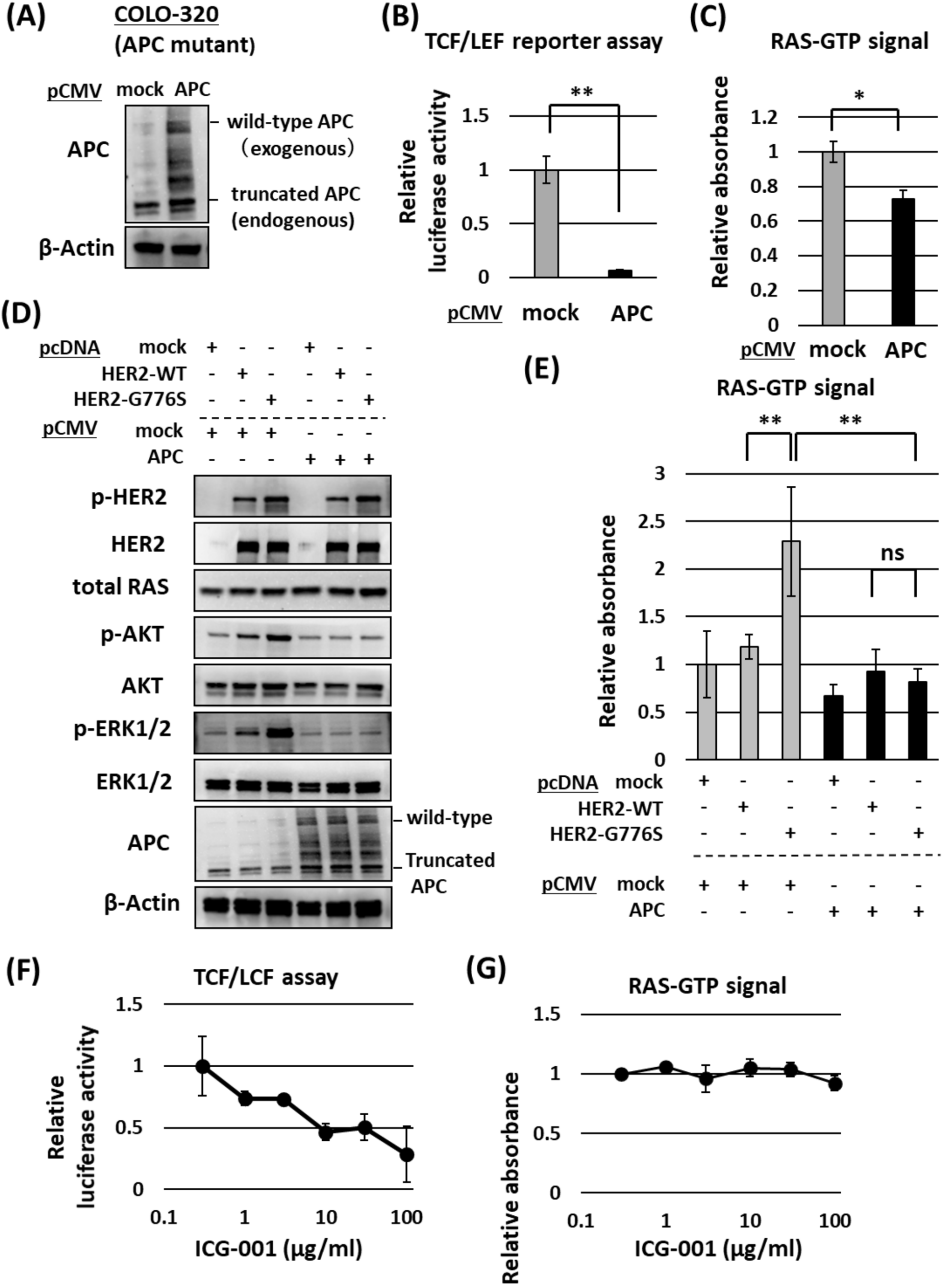
Effects of WT *APC* overexpression on the HER2 signaling pathway in COLO-320 cells (with mutant *APC*). (**A**) Confirmation of APC overexpression in cells transfected with pCMV_APC vector using Western blotting. (**B**) Activity of the Wnt/β-catenin pathway in COLO-320 cells transfected with pCMV_APC measured using the TCF/LEF luciferase reporter assay. The reporter vectors were cotransfected into COLO-320 cells along with the pCMV empty vector (mock) or pCMV-APC. The data were standardized to the mean of the activity of mock cells. **P < 0.01 vs mock (*n* = 3). (**C**) Measurement of activated RAS using the G-LISA assay performed 48 h after the transfection with pCMV. The data were standardized to the mean of the activity of cells transfected with mock vectors. *P < 0 .05 vs mock (*n* = 3) (**D**) Western blot results showing the effects of APC overexpression on the HER2 signaling pathway in COLO-320 cells. The cells were cotransfected with pcDNA_HER2-WT/G776S vectors and/or pCMV_APC vectors. Western blotting was performed 48 h after transfection. β-Actin served as a loading control. (**E**) Measurement of activated RAS using the G-LISA assay performed 48 h after transfection. The data were standardized to the mean of the activity of cells transfected with mock vectors. Two-way ANOVA: interaction *P* < 0.01, **P < 0.01 (*n* = 3). (**F**) Activity of the Wnt/β-catenin pathway in COLO-320 cells measured 24 h after addition of ICG-001 at different concentrations. The assay was performed in triplicate. (**G**) Measurement of activated RAS using the G-LISA assay in COLO-320 cells 24 h after addition of ICG-001 at different concentrations. The assay was performed in triplicate.

We investigated the interaction between APC and HER2 G776S in CRC cells (COLO-320 cells). First, we transfected the *APC* WT expression plasmid vector (pCMV-Neo-Bam APC) into COLO-320 cells with an *APC* nonsense mutation. As shown in Fig. 5A, full-length APC was detected in *APC* WT-overexpressing COLO-320 cells along with the endogenous truncated APC protein. The activity of the Wnt/β-catenin signaling pathway was markedly reduced by the expression of exogenous APC (Fig. 5B). RAS–GTP signal activity was also significantly decreased (Fig. 5C), although the amounts and phosphorylation of other proteins in the HER2–RAS–ERK pathway were not altered by the expression of exogenous APC (Supplementary Fig. 4B).

Next, we cotransfected the *APC* WT expression vector with the *HER2* WT or *HER2* G776S expression vector into COLO-320 cells. *HER2* G776S transfection increased the phosphorylation of AKT and ERK1/2 in cells without *APC* transfection, whereas their phosphorylation was not increased in cells with *APC* transfection (Fig. 5D). In COLO-320 cells, the amount of RAS–GTP was significantly increased by *HER2* G776S transfection, but was not increased when *APC* was cotransfected (Fig. 5E).

To elucidate further the effect of Wnt/β-catenin pathway activity on RAS–GTP, we cultured COLO-320 cells with ICG-001, a Wnt pathway inhibitor that antagonizes β-catenin/TCF-mediated transcription^21^. As shown in Fig. 5F, ICG-001 dose-dependently suppressed Wnt pathway activity in COLO-320 (*APC* mutant) cells but did not change the amount of RAS–GTP (Fig. 5G). These results indicate that inhibition of transcription downstream of the Wnt pathway dose not suppress the amount of RAS–GTP.

### Inhibition by afatinib of the proliferation and tumor growth of colon cancer cells harboring *HER2* G776S and *APC* mutations

**Figure 6:**
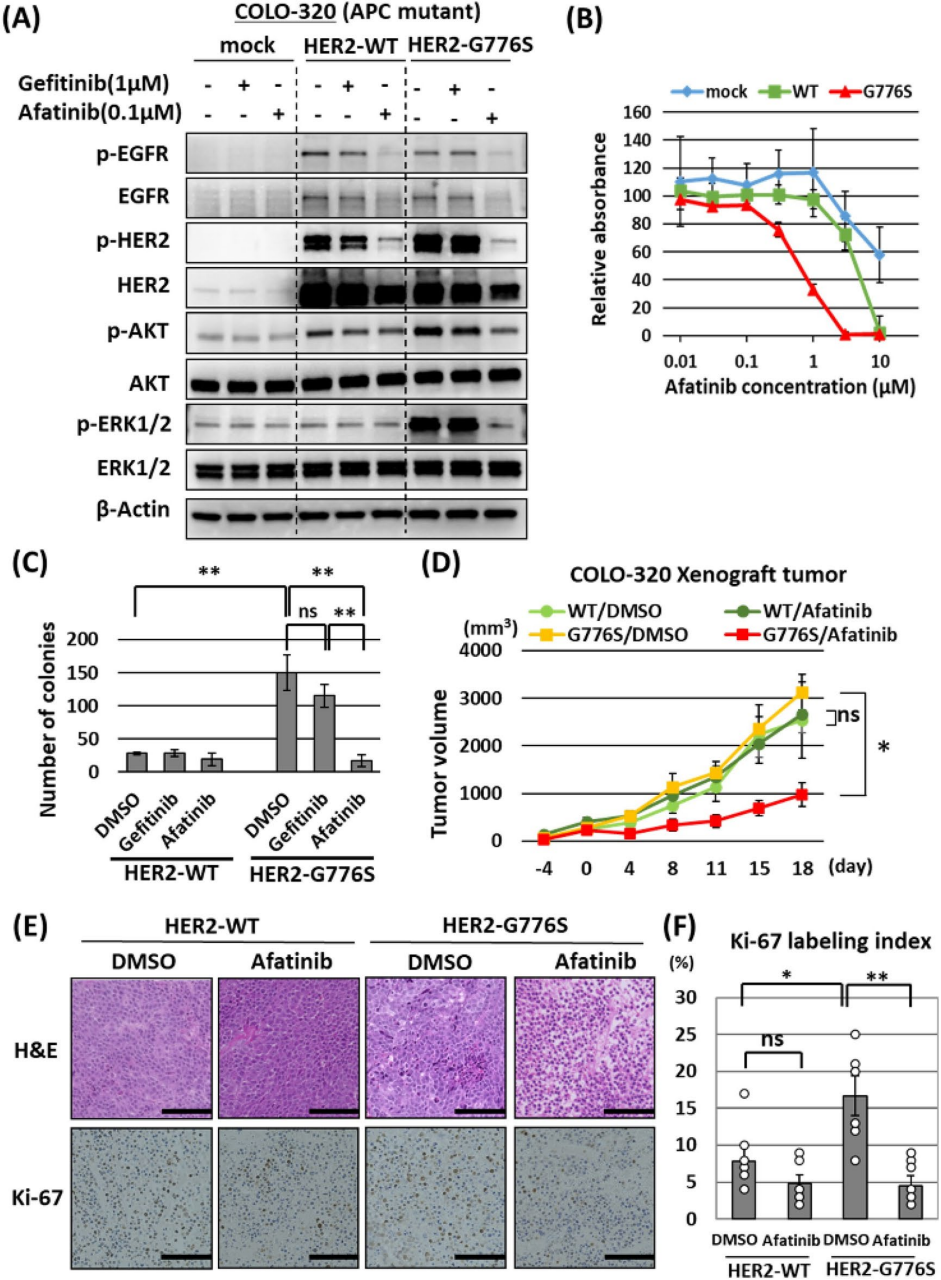
Efficacy of afatinib treatment in COLO-320 cells stably transfected with *HER2* G776S. (**A**) Western blot results show the effects of gefitinib (1 μM) or afatinib (0.1 μM) treatment in COLO-320 cells transfected with WT *HER2* or *HER2* G776S. One day after seeding, the cells were treated for 24 h. (**B**) Cell proliferation assay (WST-1 assay) results show the effects of afatinib treatment in COLO-320 cells transfected with *HER2* expression vectors. The cells were exposed to afatinib at the indicated concentrations for 24 h. (**C**) Number of colonies per well in the colony-forming assay treated with DMSO, gefitinib (1 μM) or afatinib (0.1 μM). Cells were seeded into six-well plates in triplicate and treated for 10 days. The number of colonies was counted in six random low-power fields. ns, not significant. **P < 0.01 vs DMSO treatment. (**D**) The efficacy of afatinib treatment in *HER2* WT or *HER2* G776S stably expressing COLO-320 xenograft tumors. Xenograft tumors were treated with DMSO or afatinib (25 mg/kg/day p.o.). Two-way ANOVA: interaction P < 0.01, ns, not significant. *P < 0.05 (*n* = 6). (**E**) Images of hematoxylin and eosin staining of xenograft tumor tissues in each treatment group and images of immunohistochemical staining for Ki-67 on day 18. Scale bar, 100 μm. (**F**) Rates of Ki-67-positively stained cells observed in six random fields. ns, not significant. Two-way ANOVA: interaction *P* < 0.01, *P < 0.05, **P < 0.01.

To investigate the efficacy of *HER2*-targeted therapy in colon cancer cells harboring *HER2* G776S, we examined the effect of treatment with afatinib, an irreversible pan-HER tyrosine kinase inhibitor (TKI). We investigated the cytotoxic effect of afatinib on COLO-320 cells (mutant *APC*) stably transfected with *HER2* WT or *HER2* G776S. Cells were also treated with gefitinib (EGFR TKI) for comparison. As shown in Fig. 6A, *HER2* WT or G776S transfection increased EGFR and HER2 phosphorylation in COLO-320 cells, and afatinib inhibited both EGFR and HER2 phosphorylation. By contrast, gefitinib did not inhibit phosphorylation of either protein. Afatinib strongly suppressed AKT and ERK1/2 phosphorylation in *HER2* G776S-transfected cells. Similarly, afatinib inhibited the cell viability of *HER2* G776S-transfected COLO-320 cells compared with *HER2* WT-transfected cells, but gefitinib did not alter their viability (Fig. 6B and Supplementary Fig. 5). In addition, as shown in Fig. 6C, afatinib significantly inhibited the anchorage-independent growth acquired by *HER2* G776S transfection, but gefitinib did not. By contrast, gefitinib and afatinib did not alter the anchorage-independent growth of *HER2* WT-transfected cells.

Next, we investigated the antitumor effects of afatinib using COLO-320 cells stably transfected with *HER2* WT or *HER2* G776S in a xenograft mouse model. *HER2* G776S-transfected tumors showed no significant change in their growth speed compared with WT-transfected tumors (Fig. 6D); however, the tumors had an increased Ki-67 labeling index, which is a marker for the cell cycle and tumor growth (Fig. 6E,F). Afatinib did not inhibit the growth of WT-transfected tumors, but it significantly suppressed the growth of *HER2* G776S-transfected tumors compared with the vehicle (DMSO) control group (Fig. 6D). Evaluation of the pathology revealed that *HER2* G776S-transfected tumors treated with afatinib had a sparse cell density compared with those in the vehicle control group (Fig. 6E). We confirmed that Ki-67 expression was significantly suppressed by afatinib treatment in *HER2* G776S-transfected xenograft tumors but not in *HER2* WT-transfected tumors (Fig. 6F).

## Discussion

In this study, we report on the function of the *HER2* G776S mutation, which was detected in a patient with CRC who harbored an *APC* nonsense mutation. *HER2* G776S transfection slightly increased the phosphorylation and kinase activity of HER2 but did not increase HER2 downstream signaling activity in cells with *APC* WT. We found that *HER2* G776S increased the downstream signaling activity in cells with loss of *APC* function through an increase in the amount of RAS–GTP. We also found that afatinib, a pan-HER TKI, inhibited tumor growth of *HER2* G776S-transfected COLO-320 cells with mutant *APC*.

Although most *HER2* kinase domain mutations are categorized as activating^10,17^, the kinase activity of *HER2* G776S was weaker than that of G776 > VC and insufficient to activate downstream signaling. However, when accompanied by *APC* mutations, *HER2* G776S activated downstream signaling and functioned as an oncogenic driver mutation. These results are consistent with previous reports showing that the activities of mutant *HER2* are diverse and that some of these do not result in apparent oncogenic changes^12,22^. The significance of these less active mutations is not clear, but they may acquire oncogenic potential through the coexistence of other genetic variants, such as *APC* mutations. *APC* mutations occur in more than 80% of CRCs^23^, and may have a significant impact on the role and distribution of mutant HER2 in CRC. As far as we can find, there are three cases with *HER2* G776S mutation in the COSMIC database (Fig. 1D), but these cases are not colorectal carcinoma and do not have *APC* mutation. However, we found all three cases had mutations of other genes (2, 4, and 10 genes, respectively) in the WNT/ β-catenin pathway-related gene set (KEGG_WNT_SIGNALING_PATHWAY) (Supplementary Table 2). We speculate that these cases may involve combined effects of the Wnt pathway and HER2 G776S mutation.

**Figure 7:**
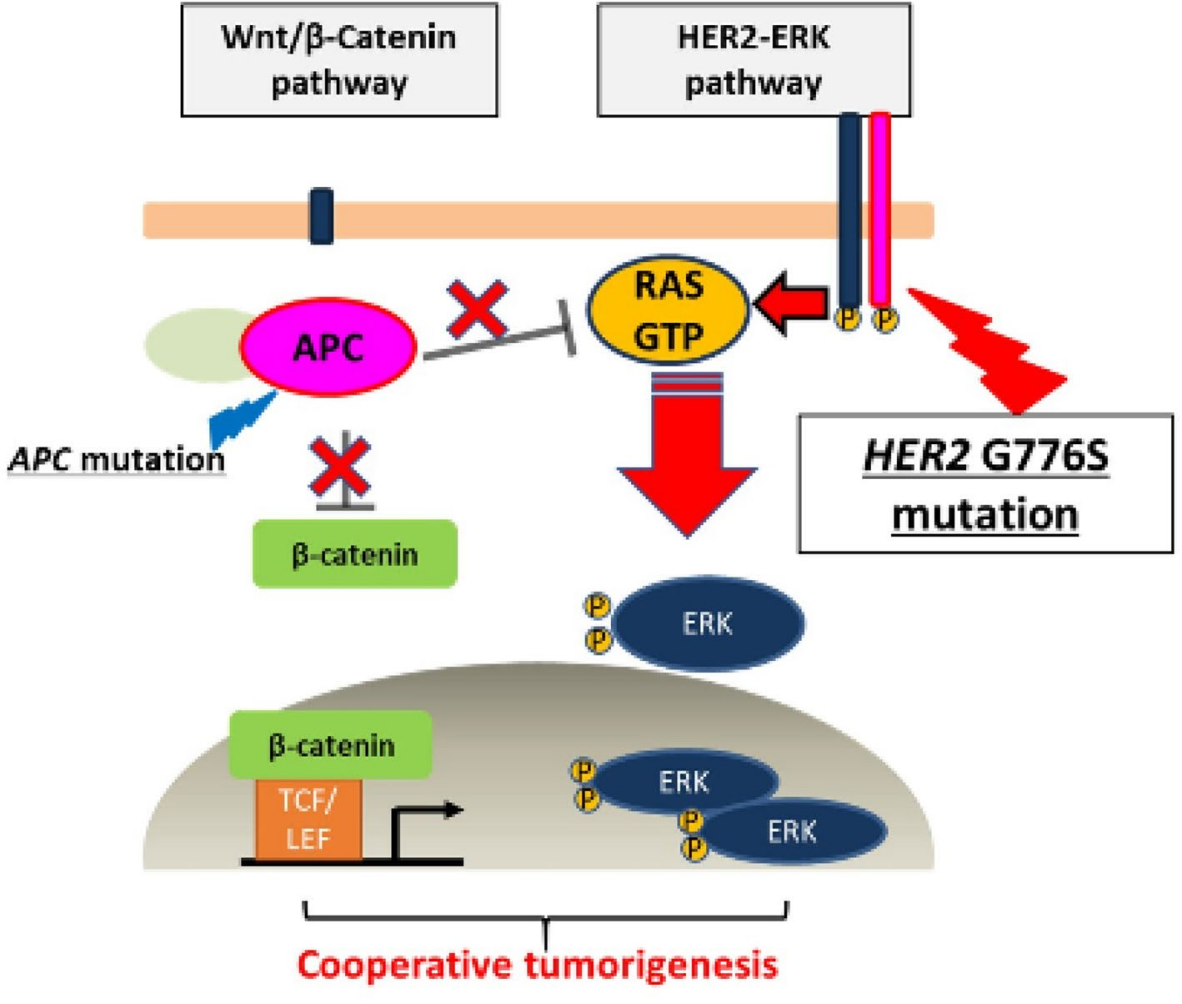
Schema of the relationship between *HER2* mutations and *APC* mutations in colorectal cancer cells. *HER2* G776S is a mutation that increases HER2 kinase activity, although the activity is weak. APC loss-of-function increases Wnt/β-catenin pathway activation but also increases the amount of RAS-GTP, which helps the *HER2* G776S mutation to activate the HER2-ERK pathway.

Although *APC* loss of function activates the Wnt pathway through the accumulation of β-catenin^24^, our results show that loss of APC function increases the amount of activated RAS (RAS–GTP) as well as the activity of the Wnt pathway. This suggests the possibility of cross talk between the HER2–RAS–ERK pathway and the Wnt/β-catenin pathway. Our data are consistent with those of previous reports showing that *APC* and *RAS* mutations affect each other through interactions between these two pathways^25,26^. In more recent studies, loss of APC function has been shown to stabilize the activated RAS, which results in increased function of the mutant RAS and leads to an early stage of colorectal carcinogenesis^27,28^. Although little has been reported on the interaction between *HER2* mutations and *APC* mutations, our findings suggest that the *HER2* G776S mutation induces significant activation of the ERK pathway via the increase in activated RAS due to loss of APC function (Fig. 7). In our study, ICG-001, a β-catenin/TCF inhibitor, reduced Wnt activity but did not affect the amount of activated RAS (Fig. 5F,G). This result suggests that the crosstalk with RAS occurs upstream of β-catenin in the Wnt pathway and that suppression downstream of the Wnt pathway alone does not inhibit ERK signaling. Further detailed studies are needed to determine whether this crosstalk is directly dependent on APC mutations and whether the crosstalk effect differs depending on the type of APC mutations.

Next, we evaluated whether this *HER2* mutation may be a therapeutic target in the treatment of CRC. In recent years, a variety of HER2-targeted therapies have attracted attention, including HER2 monoclonal antibodies, HER2 TKIs, and antibody–drug conjugates such as trastuzumab deruxtecan (T-DXd). Clinical trials have shown the efficacy of these therapies for treating in HER2-positive CRC. The HERACLES^29^ and MyPathway^30^ trials have investigated trastuzumab-based combination therapy for HER2-amplified CRC. The recently reported DESTINY-CRC01 trial^31^ showed the efficacy of T-DXd in HER2-expressing CRC. Although these trials did not evaluate HER2-mutated CRC directly, their results suggest that the various HER2-targeted therapies may be considered as future treatment strategies for HER2-mutated colorectal cancer. In this study, we examined the effect of treatment with afatinib, an irreversible pan-HER TKI, because it is expected to inhibit kinase activity increased by the G776S kinase domain mutation. Afatinib has been already approved by the US Food and Drug Administration for clinical use in non-small-cell lung carcinoma^32^ and has been reported to be effective in treating several cases of cancer with *HER2* mutation^33^.

To evaluate the effect of afatinib on CRC harboring *HER2* G776S, we established xenografts with COLO-320, which include *APC* mutation. *HER2* G776S transfection did not significantly increase tumor growth but increased the Ki-67 labeling index, a marker of cell cycle and tumor growth. Because COLO-320 cells are small and densely packed, and internal necrosis occurs as the tumor grows, there may have been little difference in the growth rate of the tumors. We found that afatinib was effective in suppressing the growth of xenograft tumors derived from *HER2* G776S-transfected COLO-320 cells. This result suggests that *HER2* G776S acted as a driver mutation in COLO-320 cells and that the cells were highly dependent on the mutated HER2 for xenograft formation. This patient was not treated with HER2-targeted therapy because the *HER2* G776S mutation was not known to be oncogenic in advance. However, we believe that this study will help to provide a rationale for targeted therapy in future cases. If effective Wnt pathway inhibitors are developed in the future, combination therapy to hit both the Wnt and HER2 pathways may be worthwhile.

Our study has some limitations. First, cells were analyzed when expressing increased levels of the mutant *HER2* and not endogenous levels. Analysis of overexpression is complicated because overexpression of *HER2* itself causes some degree of transforming activity^7^. Therefore, we compared the transforming potential between *HER2* mutants and *HER2* WT whose expression levels were maintained at the same level using cell sorting and flow cytometry. Second, this study was conducted on a minor *HER2* mutation observed in a single clinical case, and the findings may not contribute much to clinical medicine. However, clinical sequencing detects many rare mutations of unknown significance in practice. Each of them may have a small role in carcinogenesis but, as revealed in this study, some may be carcinogenic through synergistic effects and may be potential targets for therapy. We believe that our study provides information that will be relevant as clinical sequencing becomes more important in the choice of cancer treatment. Finally, the *HER2* mutation in this study was detected in the liver metastases. We were not able to examine the genetic profile of the primary site because the primary surgery was performed at another hospital several years previously and the primary site tissue was not available. However, metastases have a similar mutational profile as the primary tumor^34^^,^^35^, and we speculate that the gene profile in our study was not limited to metastatic tumors.

In conclusion, this study shows that the *HER2* G776S mutation triggers *HER2* downstream signaling activation when accompanied by *APC* loss of function and that this may be a potential therapeutic target in CRC. Our results suggest that the oncogenicity and potential targeting of *HER2* mutations in CRC should be considered in the context of *APC* function. Our findings provide novel insights into HER2 functions and strategies to target them in CRC.

## Methods

### Clinical sequencing using an NGS-based multiplex gene assay

The formalin-fixed paraffin-embedded tumor tissue of the patient was submitted to EA Genomics (Morrisville, NC) to obtain the profile using an NGS-based multiplex gene assay (OncoPrime (3); Mitsui Knowledge Industry, Tokyo, Japan) covering 215 cancer-related genes (Supplementary Table 3).

### Cell lines and investigation of their genetic features

HeLa cells were purchased from JCRB Cell Bank (Ibaraki, Japan), COLO-320 and Ba/F3 cells were purchased from Riken BioResource Center (Ibaraki, Japan), and FHC cells were purchased from ATCC (Manassas, VA). NIH/3T3 and CACO-2 cells were kindly provided by the Gastroenterology Department, Kyoto University, in April 2016. CACO-2 cells were validated using Short Tandem Repeat analysis by the Cell Line Authentication Service of Promega (Madison, WI) in March 2021. The composition of the medium for each cell is shown in Supplementary Table 4. All cells were cultured at 37 °C in a 5% CO_2_ incubator.

The genetic features of the human cell lines, HeLa, CACO-2, and COLO-320 were examined using the database Cancer Cell Line Encyclopedia (https://portals.broadinstitute.org/ccle). Because FHC cells are not available in the database, we examined their genetic features by targeted NGS using the Ion AmpliSeq Comprehensive Cancer Panel (Thermo Fisher Scientific, Waltham, MA) in accordance with the manufacturer’s instructions. Variants of genes associated with the HER2 signaling pathway and Wnt/β-catenin pathway in the cell lines are shown in Supplementary Table 1. *HER2* copy number changes were analyzed by quantitative polymerase chain reaction (PCR) using the qBiomarker Copy Number PCR Assay Kit (Qiagen, Hilden, Germany). HER2 primers (VPH117-0412044A) and reference primer set (VPH000-0000000A) were purchased from Qiagen.

### Design and construction of the expression plasmid vectors

pDONR223-ERBB2^36^, which is a plasmid vector harboring human HER2 cDNA, was a gift from William Hahn and David Root (Addgene plasmid #23888; http://n2t.net/addgene:23888; RRID:Addgene_23888). pCMV-Neo-Bam APC was a gift from Bert Vogelstein (Addgene plasmid # 16507; http://n2t.net/addgene:16507; RRID:Addgene_16507)^24^.

We established plasmid expression vectors containing human cDNA encoding full-length *HER2* wild-type (WT) and mutant *HER2* G776S. In addition, an expression vector for mutant HER2 G776 > VC, a known driver mutation in lung cancer^37^, was established as a positive control. To construct WT *HER2* expression vectors, the coding sequence of *HER2* cDNA was amplified by PCR using the pDONR223-ERBB2 as a template, and the PCR fragment was subcloned into the pcDNA3.1 vector (Thermo Fisher Scientific) using an In-Fusion HD Cloning Kit (Takara, Otsu, Japan). The resultant vectors are referred to as pcDNA_HER2-WT. To construct mutant *HER2* expression vectors, *HER2* G776S and *HER2* G776 > VC were directly induced into the pcDNA_HER2 WT vector by site-directed mutagenesis using a PrimeSTAR Mutagenesis Basal Kit (Takara) with the mutagenic primers. The resultant vectors are referred to as pcDNA_HER2 G776S and pcDNA_HER2 G776 > VC. The sequences of oligo DNAs used for vector construction (PCR primers and mutagenic primers) are shown in Supplementary Table 5.

### Transfection and establishment of stably transfected cells

The plasmids were transfected into cells using Lipofectamine LTX and Plus Reagent (Thermo Fisher Scientific) following the manufacturer’s instructions. Control cells were mock transfected with the same amount of the pcDNA or pCMV empty vector. Experiments using the transiently transfected cells were performed 48 h after transfection. To establish stably transfected cells with these plasmids, the transfected cells were cultured in 400 μg/mL hygromycin B (Nacalai Tesque, Kyoto, Japan) medium for 14–20 days. After drug selection, we aligned *HER2* expression to the same level using cell sorting on a FACSAria III flow cytometer (BD Biosciences, Franklin Lakes, NJ) using allophycocyanin-conjugated anti-human HER2 antibody (Biolegend, San Diego, CA). Seven days after cell sorting, the experiments using stably transfected cells were performed.

### Cell-based transfection assays using murine cell lines, NIH/3T3 and Ba/F3 cells

Focus formation assays were performed using NIH/3T3 cells stably transfected with pcDNA-mock, HER2- WT, G776S, or G776 > VC expression plasmid vectors. The cells (3 × 10^5^) were plated into 6 cm-diameter dishes and incubated in DMEM supplemented with 5% calf serum. Cells were fixed 12–14 days later and stained with a solution containing 30% methanol and 0.4% crystal violet. Foci larger than 1 mm in diameter were scored.

Ba/F3 transformation assay were performed using IL-3-dependent Ba/F3 cells stably transfected with the above plasmids. The cells (1 × 10^5^) were washed three times in PBS and seeded in complete medium without interleukin 3, and the number of viable cells was counted 5 days after seeding. Viability was determined by trypan blue exclusion.

### Establishment of *APC*-knockout HeLa cells using the CRISPR-Cas9 system

*APC* knockout (KO) cells were generated using LentiCRISPR v2, which was a gift from Feng Zhang (Addgene plasmid #52961; http://n2t.net/addgene:52961; RRID:Addgene_52961)^38^, following the manufacturer’s instructions. The sequences of single guide RNA (sgRNA) and oligos are listed in Supplementary Table 5. The sgRNA for *APC* KO was designed using GPP sgRNA Designer (https://portals.broadinstitute.org/gpp/public/). Cloning of the lentiCRISPR targeting *APC* was performed in the backbone of the all-in-one lentiCRISPR v2 vector, which contains the inserts Cas9 and the puromycin resistance marker. Forward and reverse oligos were mixed, annealed, and ligated with the BsmBI-digested lentiCRISPR backbone and transformed into Stbl3 chemically competent *Escherichia coli* (Invitrogen, Carlsbad, CA). Single bacterial colonies were selected and transferred to culture for the minipreparation of plasmids (QIAprep Spin Miniprep Kit; Qiagen). The established plasmids were verified by Sanger sequencing.

The resultant plasmids were transfected into HEK293T cells to produce viral particles using Trans-Lentiviral Packaging Kits (Horizon, Cambridge, UK) following the manufacturer’s instructions. HeLa cells (3 × 10^5^) were incubated in DMEM containing these viral particles and 10 μg/mL Polybrene. The plate was centrifuged at 1800 rpm at 32 °C for 1 h, and 2 days after the initial infection, the cells were refreshed with medium containing puromycin (1.0 μg/mL). After drug selection, single-cell colonies were isolated using cloning rings. Successful *APC* KO in clones was confirmed by Western blotting and TCF/LEF reporter assays.

### Kinase activity assay of WT and mutant HER2 proteins

HeLa cells were transiently transfected with pcDNA-mock, HER2 WT, G776S, or G776 > VC as mentioned above and lysed with RIPA buffer (Nacalai Tesque) 48 h after transfection. Recombinant HER2 WT and mutant proteins in the lysate were purified by immunoprecipitation using Protein A Dynabeads (Thermo Fisher Scientific) and added rabbit monoclonal anti-HER2 (29D8) antibody (Cell Signaling Technology, Danvers, MA) following the manufacturer’s instructions.

The purified proteins were eluted into elution buffer under nondenaturing conditions and incubated with Poly-(4:1 Glu, Tyr) substrate peptide (Cosmo Bio, Tokyo, Japan) along with the kinase reaction mix from the ADPsensor Universal Kinase Activity Assay Kit (BioVision, Milpitas, CA) following the manufacturers’ instructions. The fluorescent signal was measured using an Infinite F200 PRO microplate reader (Tecan Group, Ltd., Männedorf, Switzerland).

### Western blotting

The total cell lysate was extracted with lysis buffer, which is a mixture of RIPA buffer (Nacalai Tesque) and PhosSTOP phosphatase inhibitor (Sigma-Aldrich, St. Louis, MO). Western blot analysis was performed as described previously^39^. The antibodies and enhanced chemiluminescence solution are described in Supplementary Table 6. Full-length APC was detected with APC antibody (#2504) from Cell Signaling Technology, and truncated APC was detected with APC antibody (sc-9998) from Santa Cruz Biotechnology (Dallas, TX) raised against the N-terminus of APC. β-Actin was used as the loading control.

### Soft agar colony-formation assay

Anchorage-independent cell growth activity was assessed using a soft agar colony-formation assay. Base layers of growth medium containing 1.0% low melting point agarose (Lonza, Basel, Switzerland) were poured into six-well plates and allowed to solidify. Cells (2.5 × 10^4^ per well) were plated in triplicate in the top layer comprising growth medium containing 0.67% agarose. In the treatment group, gefitinib (1.0 μM), afatinib (0.1 μM), or dimethyl sulfoxide (DMSO) vehicle (0.5%) was added to the cultures. Ten days after seeding, the plates were stained with crystal violet (Sigma-Aldrich) for 1 h. Colonies measuring > 50 μm were counted using a Biorevo BZ-9000 inverted microscope (Keyence, Osaka, Japan).

### TCF/LEF activity assay

Wnt/β-catenin signaling activity was monitored using the TCF/LEF Reporter Kit (BPS Bioscience, San Diego, CA) following the manufacturer’s instructions. In brief, cells (1 × 10^4^/per well) were seeded into a 96-well plate. The next day, the TCF/LEF luciferase reporter vectors were transfected into cells, and the cells were incubated for 48 h. Cells were harvested, lysed, and analyzed using the Dual-Luciferase Reporter Assay System (Promega) to measure luciferase activity.

### RAS–GTP assay

To examine RAS activity, we measured the amount of RAS–GTP from cell lysates using an RAS Activation G-LISA Kit (Cytoskeleton, Denver, CO) according to the manufacturer’s instruction. The signal was measured using an Infinite 200 PRO multiplate reader (Tecan Group Ltd.) at a wavelength of 490 nm. The results are expressed as the fold increase in activity compared with the control group.

### Cell proliferation assay

Cell proliferation was quantified using a WST-1 assay (Roche Applied Science, Penzberg, Germany) according to the manufacturer’s instructions. Cells (1 × 10^4^ cells/well) were seeded into 96-well plates. All data were obtained using an Infinite 200 PRO multiplate reader (Tecan Group Ltd.) at a wavelength of 450 nm.

### Xenograft models and in vivo afatinib treatment

Six-week-old BALB/cAJcl-*nu/nu* mice (CLEA Japan, Tokyo, Japan) were used for in vivo assays. To establish xenograft tumors derived from COLO-320 cells stably transfected with pcDNA-mock, *HER2*-WT, or *HER2*-G776S, each cell line (2 × 10^6^ cells) was suspended in 50% Matrigel (BD Biosciences) and injected subcutaneously into the flanks of the mice. Tumor volume was measured with calipers every 3 or 4 days and was calculated using the empirical formula: tumor volume = ½ × [(the shortest diameter)^2^ × (the longest diameter)]. When the tumor volume was about 100 mm^3^, mice were randomly allocated to two groups (*n* = 6 in each group) that received either vehicle or 25 mg⁄kg afatinib orally by gavage once daily for 6 days per week. After 3 weeks of treatment, the mice were sacrificed, and the tumor pathology was evaluated. Ki-67-positive cells were scored by counting at least 1000 cells per low-power field under a Biorevo BZ-9000 light microscope (Keyence).

All animal experiments conformed to the relevant regulatory standards and were approved by the Institutional Animal Care and Use Committee of Kyoto University (Med Kyo 17253). Isoflurane (Fujifilm Wako pure chemical, Osaka, Japan) was used to anesthetize the mice. This study was conducted in accordance with the ARRIVE guidelines.

### Statistics

All statistical analyses were performed using JMP 15 (SAS Institute Inc., Cary, NC). Two-tailed Student’s t tests were used to compare data between two groups, and One-way ANOVA and post-hoc Tukey Honest Significant Difference (HSD) tests were used to analyze three or more groups, unless otherwise indicated. The interaction between two factors was assessed using two-way ANOVA. Error bars represent ± SD. P < 0.05 was considered to be significant.

### Ethics approval and consent to participate

This study has been approved by the research ethics committee of Kyoto University (G1182-1). Written informed consent was obtained from the patient for participation in this study. All methods were performed in accordance with the relevant guidelines and regulations.

## Supplementary Information

Supplementary Information.

## Data Availability

The sequencing data in this study have been deposited into the database of the DNA Data Bank of Japan (https://www.ddbj.nig.ac.jp/index-e.html) under accession number LC684105.
