# Supplementary material for: HER2 G776S mutation promotes oncogenic potential in colorectal cancer cells when accompanied by loss of APC function
Source: Sci Rep. 2022 Jun 2;12:9213. doi: 10.1038/s41598-022-13189-y (PMC9163061; doi:10.1038/s41598-022-13189-y)
Supplement: Supplementary file 1 — Supplementary Information. [file 41598_2022_13189_MOESM1_ESM.docx]

**Manuscript title: *HER2* G776S mutation promotes oncogenic potential in colorectal cancer cells when accompanied by loss of *APC* function**

Yosuke Mitani^1^, Shinya Ohashi^1^, Osamu Kikuchi^1^, Yukie Nakai^1^, Tomomi Ida^1^, Ayaka Mizumoto^1^, Yoshihiro Yamamoto^1^, Tomoki Saito^1^, Shigeki Kataoka^1^, Junichi Matsubara^1^, Atsushi Yamada^1^, Masashi Kanai^1^, Shigemi Matsumoto^1^, Hiroaki Sakai^2^, Kiyotsugu Yoshikawa^3^, Eijiro Nakamura^4^, and Manabu Muto^1,*^

^1^Department of Therapeutic Oncology, Kyoto University Graduate School of Medicine, 54 Shogoin Kawahara-cho, Sakyo-ku, Kyoto 606-8507, Japan

^2^ DSK Project, Medical Innovation Center, Kyoto University Graduate School of Medicine, 54 Shogoin Kawahara-cho, Sakyo-ku, Kyoto 606-8507, Japan

^3^ Department of Clinical Pharmacy Faculty of Pharmaceutical Sciences, Doshisha Women’s College of Liberal Arts, W604, Keisui-kan, 97-1 Minami-hodate Kodo, Kyotanabe City, Kyoto 610-0395, Japan

^4^ Department of Urology, National Cancer Center Hospital, 5-1-1 Tsukiji, Chuo-ku, Tokyo104-0045, Japan

* To whom correspondence should be addressed. E-mail: [mmuto@kuhp.kyoto-u.ac.jp](mailto:mmuto@kuhp.kyoto-u.ac.jp)

**Supplementary Information**

Supplementary Figure 1: Copy number analysis of HER2 using qPCR.

Supplementary Figure 2: Structure of *HER2* WT and mutant *HER2* expression plasmid vectors.

Supplementary Figure 3: Cell proliferative potential of *HER2* G776S transfected colon cells.

Supplementary Figure 4: The effect of APC-KO or APC-overexpression on HER2-ERK pathway.

Supplementary Figure 5: Efficacy and IC_50_ of afatinib for colon cells transfected with *HER2* G776S.

Supplementary Table 1. Genetic characteristics of colon cell lines and HeLa cells.

Supplementary Table 2: Genetic variants related to the Wnt /β-catenin pathway in three patients with HER2 G776S mutation.

Supplementary Table 3. Target genes of the comprehensive genomic analysis.

Supplementary Table 4: The medium and supplements used in this study.

Supplementary Table 5: Sequence of primers and oligos.

Supplementary Table6: Antibodies, reagents and equipment used in Western blotting.

Full-length Western blot images.


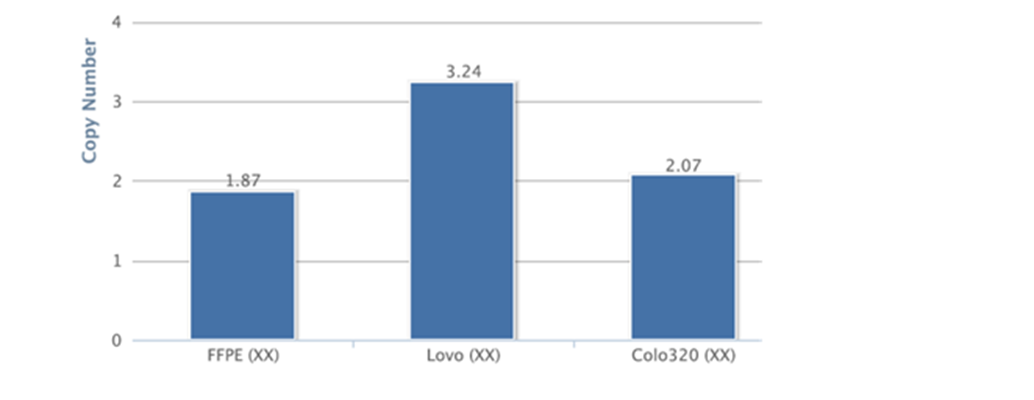


**Supplementary Figure 1: Copy number analysis of *HER2* using qPCR.** genomic DNA extracted from FFPE was analyzed. Lovo and Colo-320 cells are listed for comparison.


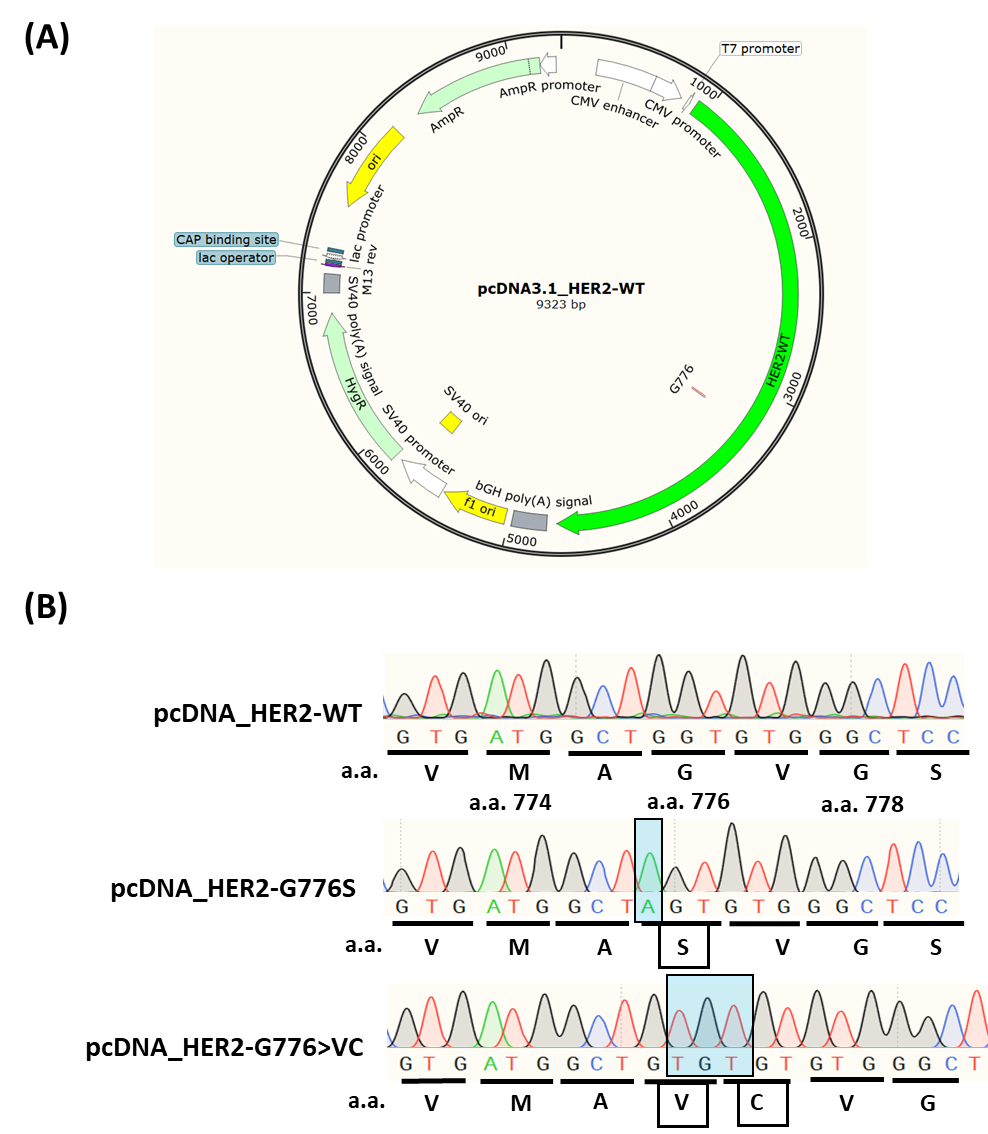


**Supplementary Figure 2: Structure of *HER2* WT and mutant *HER2* expression plasmid vectors.** (A) Genetic map and organization of pcDNA_HER2-WT. The circular image was generated with SnapGene Viewer (v. 5.1.; GSL Biotech LLC, San Diego, CA). (B) DNA Sequence of *HER2* WT, G776S, and G776>VC. The bases generated by mutation and insertion are surrounded by squares.


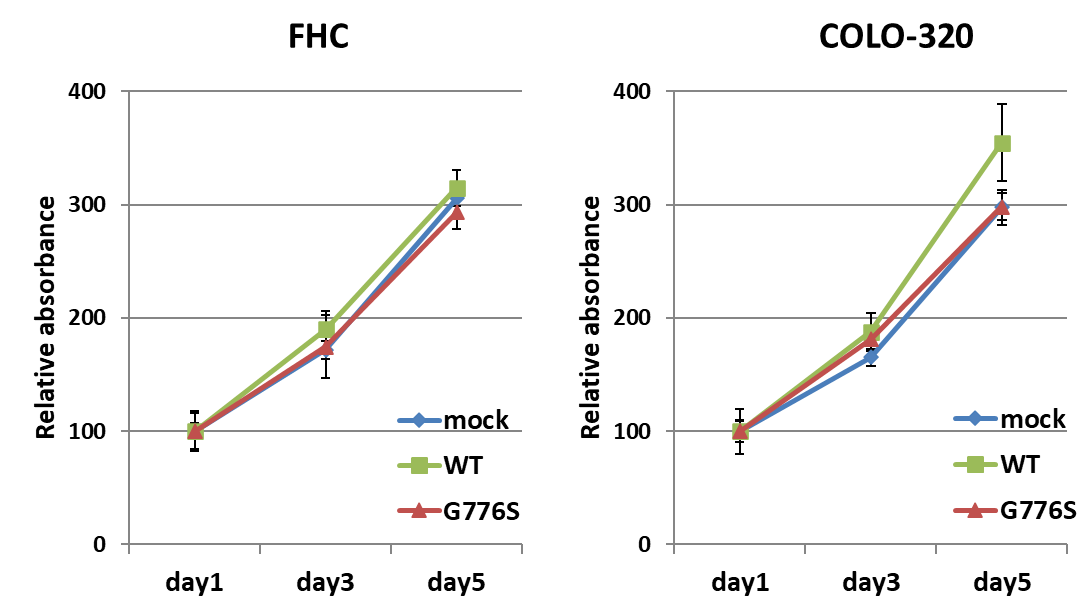


**Supplementary Figure 3: Cell proliferative potential of *HER2* G776S transfected colon cells.** FHC and COLO-320 cells stably transfected with mock or the HER2 expression vectors were seeded in 96 wells (1×10^4^ cells per well) with complete medium. Their cell proliferation was evaluated by WST-1 assay on days 1, 3 and 5, respectively (n=6).


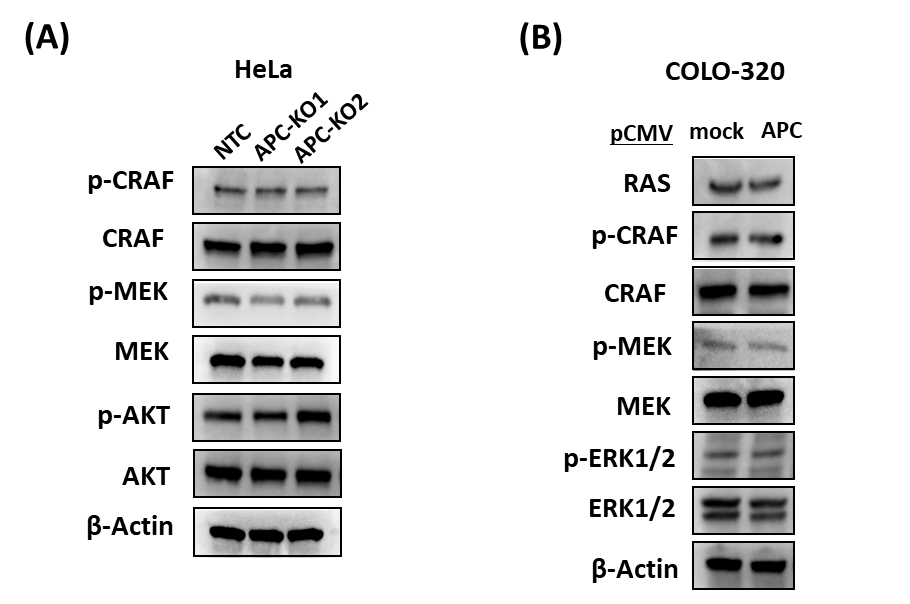


**Supplementary Figure 4: The effect of APC-KO or APC-overexpression on HER2-ERK pathway.** Phosphorylation and expression of the HER2 downstream signaling in APC-KO HeLa cells (A) or APC-expressing COLO-320 cells (B).


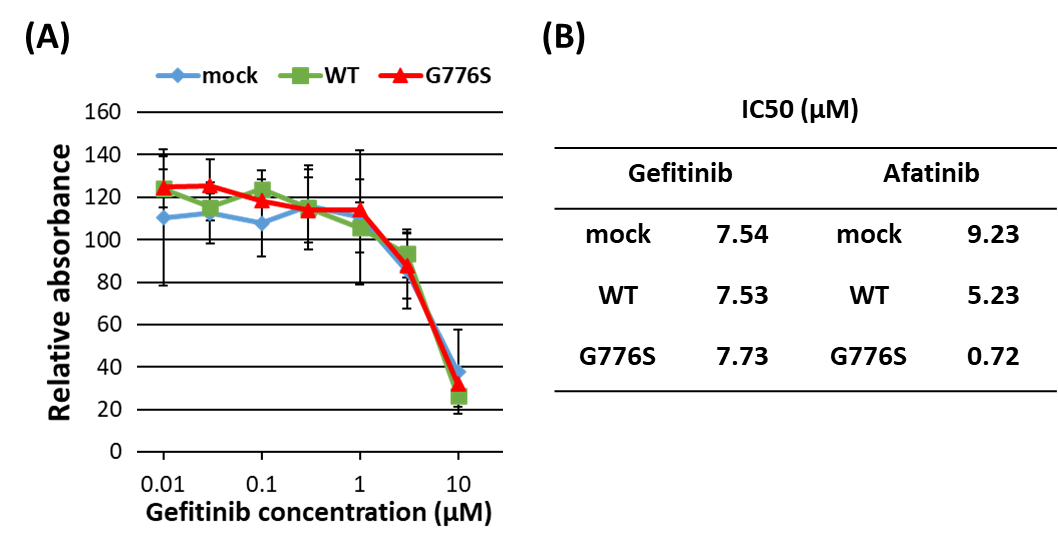


**Supplementary Figure 5:** **Efficacy and IC_50_ of afatinib for colon cells transfected with *HER2* G776S.** COLO 320 cells (mutant APC) transfected with an empty vector, *HER2* WT, or *HER2* G776S were treated with afatinib or gefitinib for 24 h and the IC_50_ values were determined using a WST-1 cell proliferation assay (Roche Applied Science, Penzberg, Germany) according to the manufacturer’s instructions.

**Supplementary Table 1. Genetic characteristics of colon cell lines and HeLa cells**

|  |  | **ERBB family** | | | **ERBB downstream signaling** | | | | **Wnt/β-catenin pathway** | | **Others** |
| --- | --- | --- | --- | --- | --- | --- | --- | --- | --- | --- | --- |
| **Cell name** | **Origin** | ***EGFR*** | ***HER2*** | ***HER3*** | ***KRAS*** | ***NRAS*** | ***BRAF*** | ***PIK3CA*** | ***APC*** | ***CTNNB1*** | ***TP53*** |
| **HeLa** | cervical cancer | - | - | - | - | - | - | - | - | - | - |
| **FHC** | colon normal | - | - | - | - | - | - | - | - | - | - |
| **Caco-2** | colon cancer |  |  |  |  |  |  |  | Q1367* | G245A | C135F  E204* |
| **COLO-320** | colon cancer | - | - | - | - | - | - | - | S811* | - | R248W |
| HCT-116 | colon cancer | - | - | Q261* | G13D | - | - | H1047R | - | S45del | - |
| HT-29 | colon cancer | - | - | - | - | - | V600E | P449T | E853* | - | R273H |
| **The patient** | colon cancer | - | G776S | - | - | - | - | - | S583* | - | G245D |

The genetic features of cell lines except FHC were examined using the database Cancer Cell Line Encyclopedia (https://portals.broadinstitute.org/ccle). FHC cells was examined their genetic features by targeted NGS. HCT-116 and HT-29 were added to the analysis for comparison. For reference, the clinical sequencing data of the patient detected HER2-G776S are appended at the bottom of the table.

**Supplementary Table 2: Genetic variants related to the Wnt /β-catenin pathway in three patients with HER2 G776S mutation.**

There are three cases with HER2 G776S mutation in the COSMIC database. The Wnt /β-catenin pathway-related genetic variants of the three cases were described with reference to the WNT/ β-catenin pathway related gene set (KEGG_WNT_SIGNALING_PATHWAY) downloaded from Gene Set Enrichment Analysis (GSEA: https://www.gsea-msigdb.org/gsea/index.jsp).

**Supplementary Table 3. Target genes of the comprehensive genomic analysis.**

| ABL | BLM | CRLF2 | ESR1 | H3F3A | MAP3K1 | NFE2L2 |
| --- | --- | --- | --- | --- | --- | --- |
| PIK3R2 | SETD2 | TP53 | ABL2 | BRAF | CSF1R | EZH2 |
| HNF1A | MAPK1 | NOTCH1 | PIK3R5 | SF3B1 | TP63 | ACVR1B |
| BRCA1 | CTNNA1 | FAM123B | HRAS | MDM2 | NOTCH2 | PMS1 |
| SMAD2 | TP73 | AKT1 | BRCA2 | CTNNB1 | FANCA | IDH1 |
| MDM4 | NOTCH3 | PMS2 | SMAD3 | TPMT | AKT2 | BTK |
| CYP1A2 | FBXW7 | IDH2 | MED12 | NOTCH4 | PPP2R1A | SMAD4 |
| TRAF7 | AKT3 | CARD11 | CYP2C19 | FGFR1 | IGF1R | MEN1 |
| NPM1 | PRDM1 | SMARCA4 | TSC1 | ALK | CASP8 | CYP2C9 |
| FGFR2 | IGF2R | MET | NRAS | PTCH1 | SMARCB1 | TSC2 |
| APC | CBL | CYP2D6 | FGFR3 | IKZF1 | MITF | NTRK1 |
| PTCH2 | SMO | TSHR | AR | CCND1 | DAXX | FGFR4 |
| IL7R | MLH1 | NTRK2 | PTEN | SOCS1 | TYMS | ARAF |
| CCND2 | DDR2 | FLT1 | INSR | MLL | NTRK3 | PTPN11 |
| SRC | U2AF1 | ARID1A | CCND3 | DNMT3A | FLT3 | JAK1 |
| MPL | PALB2 | RAD50 | SRSF2 | UGT1A1 | ARID1B | CCNE1 |
| DPYD | FLT4 | JAK2 | MRE11A | PARP1 | RAD51 | STAG2 |
| VHL | ASXL1 | CDC73 | EGFR | FOXL2 | JAK3 | MSH2 |
| PAX5 | RAF1 | STAT1 | VKORC1 | ATM | CDH1 | EP300 |
| G6PD | KDM6A | MSH6 | PBRM1 | RB1 | STAT3 | WRN |
| ATR | CDK4 | ERBB2 | GATA1 | KDR | MTHFR | PDGFRA |
| RET | STK11 | WT1 | ATRX | CDK6 | ERBB3 | GATA2 |
| KIT | MTOR | PDGFRB | RICTOR | SUFU | XPC | AURKA |
| CDKN2A | ERBB4 | GATA3 | KLF4 | MYC | PDK1 | RNF43 |
| TERT | XRCC1 | AURKB | CDKN2B | ERCC1 | GLI1 | KRAS |
| MYCN | PGR | ROS1 | TET2 | AXIN1 | CEBPA | ERCC2 |
| GNA11 | MAML1 | MYD88 | PHF6 | RPTOR | TGFBR2 | BAP1 |
| CHEK1 | ERCC3 | GNAQ | MAP2K1 | NBN | PIK3CA | RSPO2 |
| TNFAIP3 | BCL2 | CHEK2 | ERG | GNAS | MAP2K2 | NF1 |
| PIK3CG | RSPO3 | TOP1 | BCOR | CREBBP | ERRFI1 | GRIN2A |
| MAP2K4 | NF2 | PIK3R1 | RUNX1 | TOP2A |  |  |

**Supplementary Table 4: The medium and supplements used in this study.**

| Cell line | Medium | Supplements |
| --- | --- | --- |
| HeLa | DMEM | 10% fetal bovine serum  100 μg/mL of streptomycin and 100 units/mL of penicillin |
| FHC | 1:1 mixture of DMEM and F-12 | 25 mmol/L HEPES  10 ng/mL cholera toxin  0.005 mg/mL insulin  0.005 mg/mL transferrin  100 ng/mL hydrocortisone  10% fetal bovine serum  100 μg/mL of streptomycin and 100 units/mL of penicillin |
| Caco-2 | MEM | 20% fetal bovine serum  0.1μM Non-Essential Amino Acids (NEAA)  100 μg/mL of streptomycin and 100 units/mL of penicillin |
| COLO-320 | RPMI-1640 | 10% fetal bovine serum  100 μg/mL of streptomycin and 100 units/mL of penicillin |
| NIH/3T3 | DMEM | 10% fetal bovine serum  100 μg/mL of streptomycin and 100 units/mL of penicillin |
| Ba/F3 | RPMI-1640 | 10% fetal bovine serum  100 μg/mL of streptomycin and 100 units/mL of penicillin  10 ng/ml IL-3 |

| Product | Company |
| --- | --- |
| DMEM | Thermo Fisher Scientific, Waltham, MA, USA |
| 1:1 mixture of DMEM and F-12 | Thermo Fisher Scientific |
| MEM | Thermo Fisher Scientific |
| RPMI-1640 | Thermo Fisher Scientific |
| NEAA | Fujifilm Wako, Osaka, Japan |
| 10% fetal bovine serum | Thermo Fisher Scientific |
| Penicillin-Streptomycin | Thermo Fisher Scientific |
| IL-3 | Pepro Tech, Cranbury, NJ, USA |
| Cholera toxin | Fujifilm Wako |
| Insulin | Sigma-Aldrich, St. Louis, MO, USA |
| Transferrin | Sigma-Aldrich |
| Hydrocortisone | Sigma-Aldrich |

**Supplementary Table 5: Sequence of primers and oligos.**

| Construct |  | Primer name | Sequence |
| --- | --- | --- | --- |
| HER2 cording sequence | Sense | HER2_F | GCCACTGTGCTGGATTCACACTGGCACGTCCAGAC |
|  | antisense | HER2_R | GTTTAAACTTAAGCTTGCCACCATGGAGCTGGCGGCC |
| Mutagenesis G776S | sense | G776S_F | TGATGGCTAGTGTGGGCTCCCCATATGTCTCCC |
|  | antisense | G776S_R | CCACACTAGCCATCACGTATGCTTCGTCTAAGA |
| Mutagenesis G776>VC | sense | G776>VC_F | ATGGCTGTGTGTGTGGGCTCCCCATATG |
|  | antisense | G776>VC_R | CACACACACAGCCATCACGTATGCTT |
| sgRNA targeting APC |  |  | CCCGGCTTCCATAAGAACGG |
| sgRNA context sequence |  |  | CCTTCCCGGCTTCCATAAGAACGGAGGGAC |
| Forward oligo |  |  | CACCGCCCGGCTTCCATAAGAACGG |
| Reverse oligo |  |  | AAACCCGTTCTTATGGAAGCCGGGC |

**Supplementary Table 6: Antibodies, reagents and equipment used in Western blotting.**

The following from Cell Signaling Technology (CST), Danvers, MA, USA.

| **Primary antibodies** | **Concentration** | **Serial number (CST)** |
| --- | --- | --- |
| EGFR | 1:1000 | #4267 |
| phospho- (p-)EGFR (Tyr1068) | 1:1000 | #3777 |
| HER2 | 1:1000 | #2242 |
| p-HER2 (Tyr1221/1222) (6B12) | 1:1000 | #2243 |
| Akt (C67E7) | 1:1000 | #4691 |
| p-Akt (Ser473) (D9E) | 1:2000 | #4060 |
| RAS (27H5) | 1:1000 | #3339 |
| MEK 1/2 (D1A5) | 1:1000 | #8727 |
| p-MEK (Ser217/221) | 1:1000 | #9154 |
| c-RAF (D4B3J) | 1:1000 | #53745 |
| p-c-RAF (Ser338) | 1:1000 | #9427 |
| p44/p42 MAPK | 1:1000 | #9102 |
| p-p44/p42 MAPK (Thr202/Tyr204) (D13.14.4E) | 1:2000 | #4370 |
| APC | 1:1000 | #2504 |
| β-actin (HRP conjugate) (13E5) | 1:3000 | #5125 |
| **Secondary antibodies** | **Concentration** | **Serial number (CST)** |
| Anti-rabbit, IgG, HRP-linked | 1:2000 | #7074 |
| Anti-mouse IgG, HRP-linked | 1:2000 | #7076 |

| **Primary antibodies** | **Concentration** | **Company** |
| --- | --- | --- |
| APC ( sc-9998) | 1: 200 | Santa Cruz Biotechnology, Dallas, TX, USA |

| **Enhanced chemi luminescence** | **Company** |
| --- | --- |
| SuperSignal West FemtoMaximum Sensitivity Substrate | Thermo Fisher Scientific, Waltham, MA, USA |
| **Imaging analyzer** | **Company** |
| ChemiDoc Touch Imaging System | Bio-Rad Laboratories, Hercules, CA, USA |

**Full-length Western blot images**

**
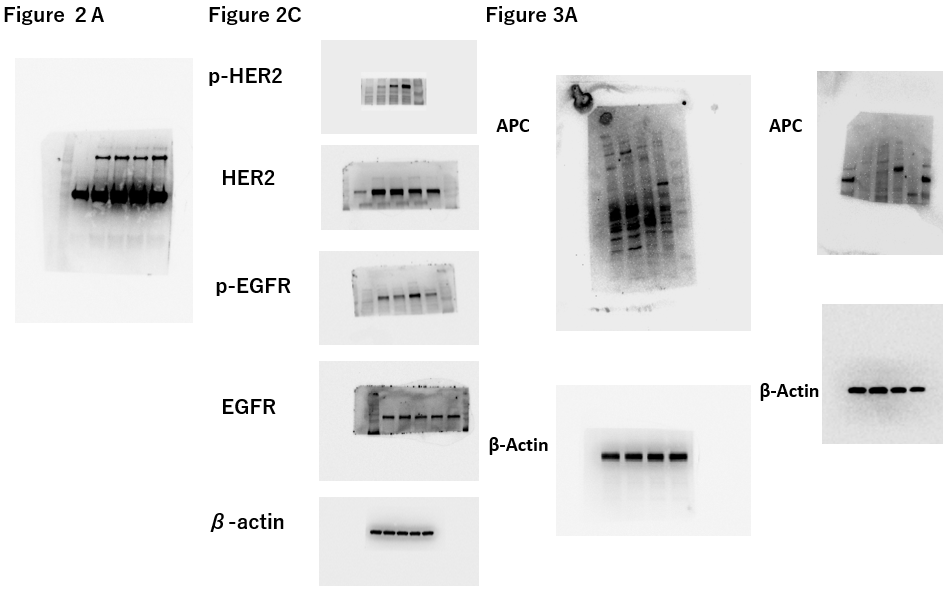
**


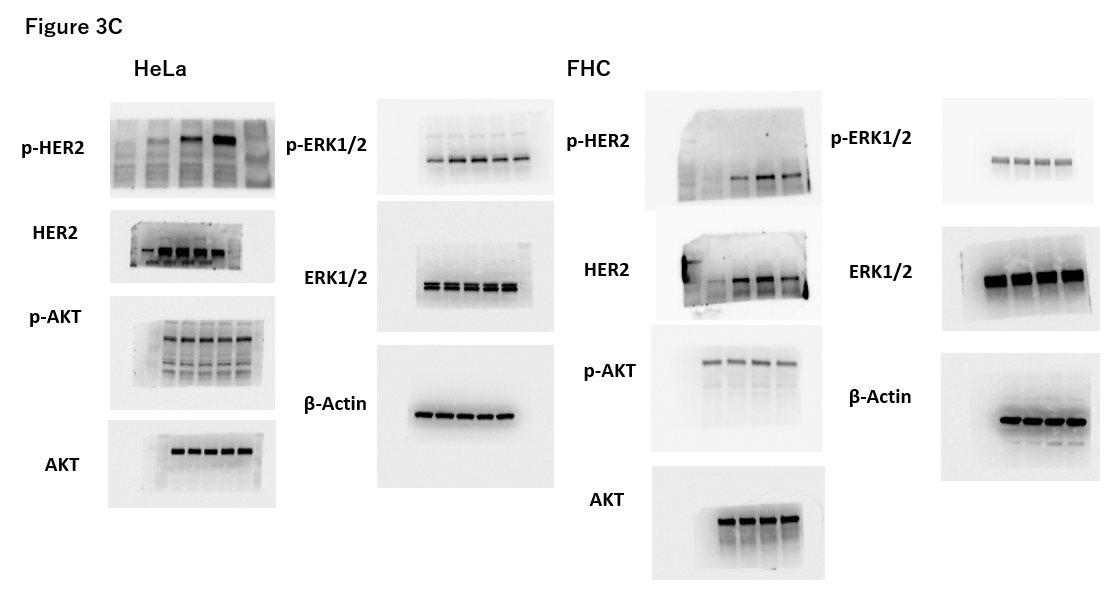

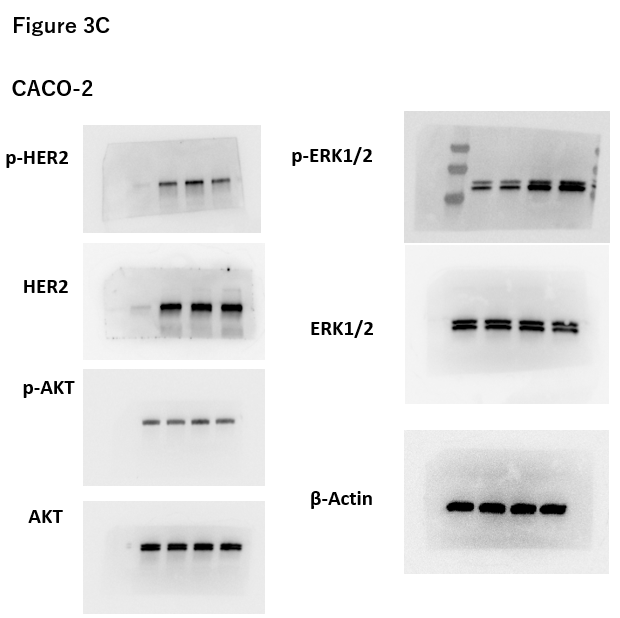

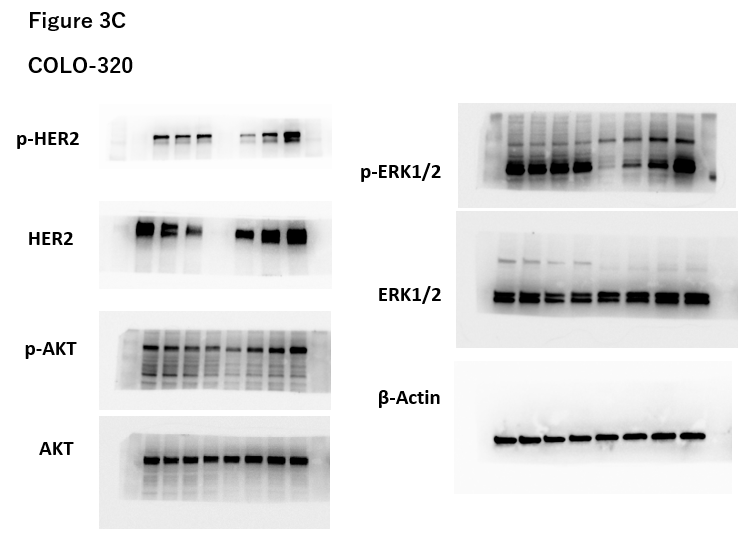

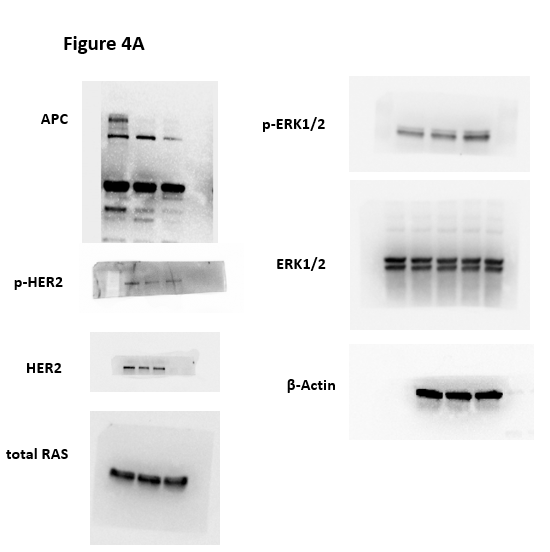

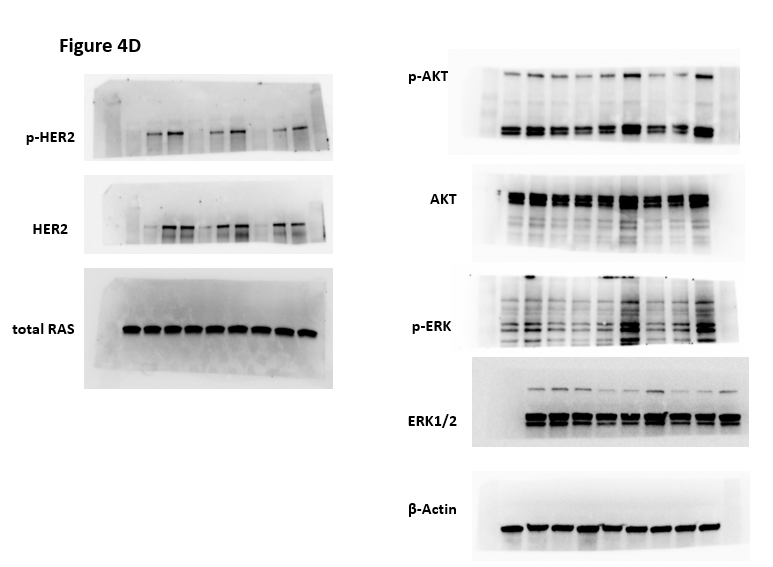

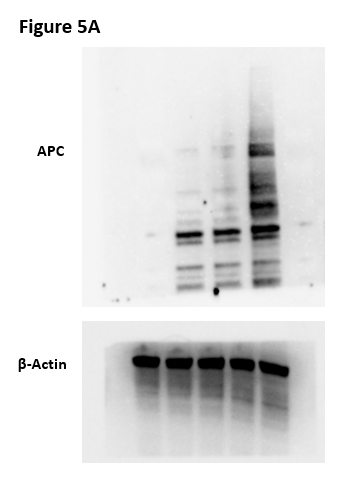

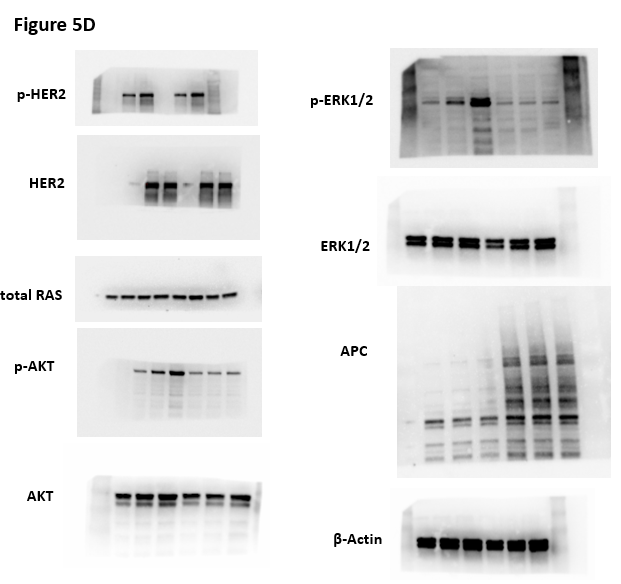

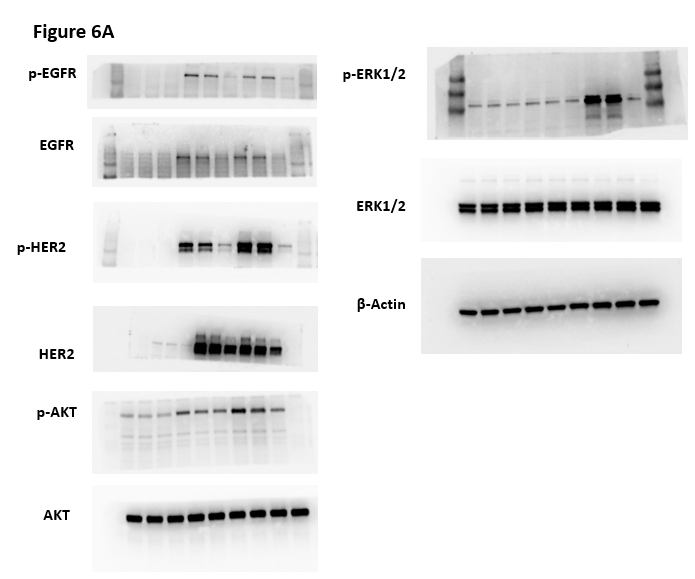


**
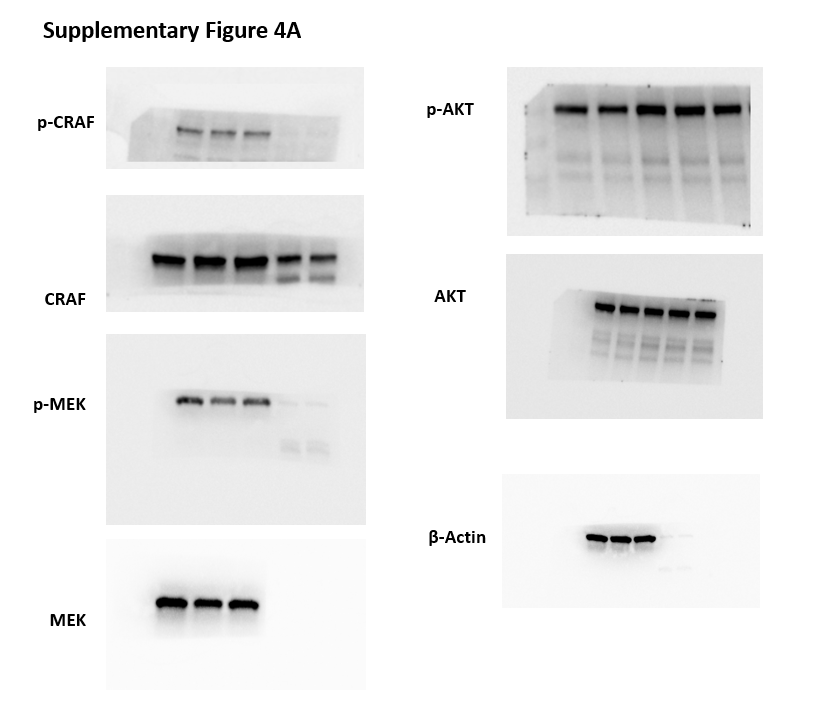
**

**
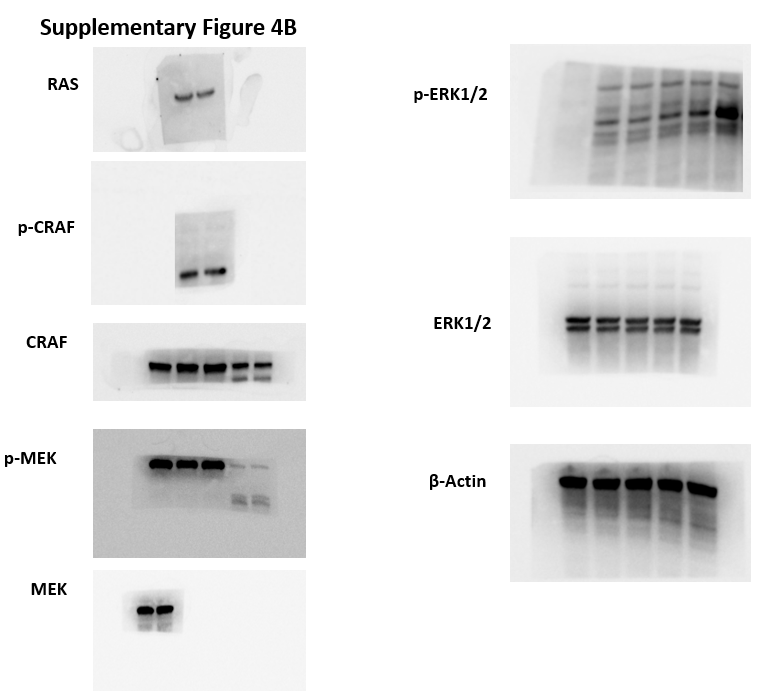
**
